# A concise mathematical description of signal transformations across the hippocampal apical CA3 to CA1 dendritic response

**DOI:** 10.3389/fncir.2025.1545031

**Published:** 2026-02-12

**Authors:** Sandra Gattas, Aliza A. Le, Javad Karimi Abadchi, Ben Pruess, Rohit Amba, Yanning Shen, A. Swindlehurst, Michael A. Yassa, Gary Lynch

**Affiliations:** 1 Department of Electrical Engineering and Computer Science, University of California, Irvine, Irvine, CA, United States; 2 Center for the Neurobiology of Learning and Memory, University of California, Irvine, Irvine, CA, United States; 3 Department of Anatomy and Neurobiology, University of California, Irvine, Irvine, CA, United States; 4 Department of Psychiatry, Douglas Hospital Research Centre, McGill University, Montreal, QC, Canada; 5 Department of Neurobiology and Behavior, University of California, Irvine, Irvine, CA, United States

**Keywords:** hippocampus, CA3, CA1, synaptic communication, Volterra series, system identification

## Abstract

The synapse is the fundamental unit of communication in the nervous system. Determining how information is transferred across the synaptic interface is one of the most complex endeavors in neuroscience, owing to the large number of contributing factors and events. An approach to solving this problem involves collapsing across these complexities to derive concise mathematical formulas that fully capture the governing dynamics of synaptic transmission. We investigated the feasibility of deriving such a formula – an input-output transformation function for the CA3 to CA1 node of the hippocampus – using the Volterra expansion technique for non-linear system identification. The timecourse of the fEPSP in the apical dendrites of mouse brain slices was described with >94% accuracy by a 2nd order equation that captured the linear and non-linear influence of past inputs on current outputs. This function generalized to cases not included in its derivation and uncovered previously undetected timing rules. The basal dendrites expressed a substantially different transfer function and evidence was obtained that, unlike the apical system, a 3rd order system or higher will be needed for complete characterization. At scale, the approach will also provide information needed for the construction of biologically realistic models of brain networks.

## Introduction

Mathematical characterization of the composite synaptic response generated by small collections of input axons is a critical step in understanding communication among brain subsystems. Efforts in this direction encounter a series of challenges. The cortical telencephalon operates with a diverse set of signaling patterns embodied in spike patterns, which requires synaptic descriptions to include a very large number of input possibilities. Relatedly, such temporal patterns engage an extraordinarily diverse array of cellular and concomitant physiological events with different time courses on both sides of the synapse (Bellocchio et al., 1998; Dittman and Ryan, 2019; Edwards, 2007; Jackman et al., 2016; Miki et al., 2016, 2018; Wang et al., 2014; Zamponi and Currie, 2013). Past responses generated in response to past inputs thus have a strong influence on current ones as exemplified in the well-studied frequency facilitation effect (Jackman and Regehr, 2017; Katz and Miledi, 1968). The description problem is further complicated by the presence of feedforward inhibitory neurons that are engaged by an active input and innervate the target dendrites for that input. This results in responses that are admixtures of monosynaptic and disynaptic events with the latter acting to shunt currents generated by the former (Schulz et al., 2018). In all, the seemingly simple potentials recorded in conventional experiments using repetitive stimulation reflect a large array of interacting variables.

The present study investigated the feasibility of capturing the size and waveform of synaptic responses in a concise mathematical formulation that accommodates time-varying input patterns. The experimental strategy followed an engineering approach referred to as “system identification” that aims at accurately characterizing all possible operations performed by an unknown system (i.e., a black box). Work of this kind, using inputs sufficient to tap into the system’s full operational range, shows that it is possible to obtain a unique solution for input-output transformations – a single formula or transfer function that predicts outputs to arbitrary inputs – for electronic circuits. System identification has also been used to capture dynamics of different biological systems and across species (Marmarelis, 2004). The experimental question was thus whether system identification can also be used to describe axo-dendritic synaptic dynamics despite the immense complexity of such a biological system.

Beyond its importance for characterizing the manner in which variations in afferent patterns are differentially translated into dendritic output, an accurate transfer function would provide a novel and precise means for addressing widely studied issues in neuroscience. By design, the approach samples the entirety of responses rather than single measures such as slope or amplitude. This along with the above noted use of a full range of time-varying inputs, as opposed to stereotyped stimulation patterns, result in a new level of sensitivity for assessing the effects of pharmacological and genetic manipulations. A predictive formula would as well enable quantitative tests for differences between synaptic populations in the processing of realistic input patterns, an issue that is of vital importance to the construction of network models. The work reported here accordingly attempted to develop transfer functions for both the apical and basal dendrites of hippocampal field CA1, one of the most intensively studied brain regions.

## Results

### Signal transformation in CA1 apical dendrites

#### Using Volterra series expansion technique to identify the CA1 apical dendritic operations

**FIGURE 1:**
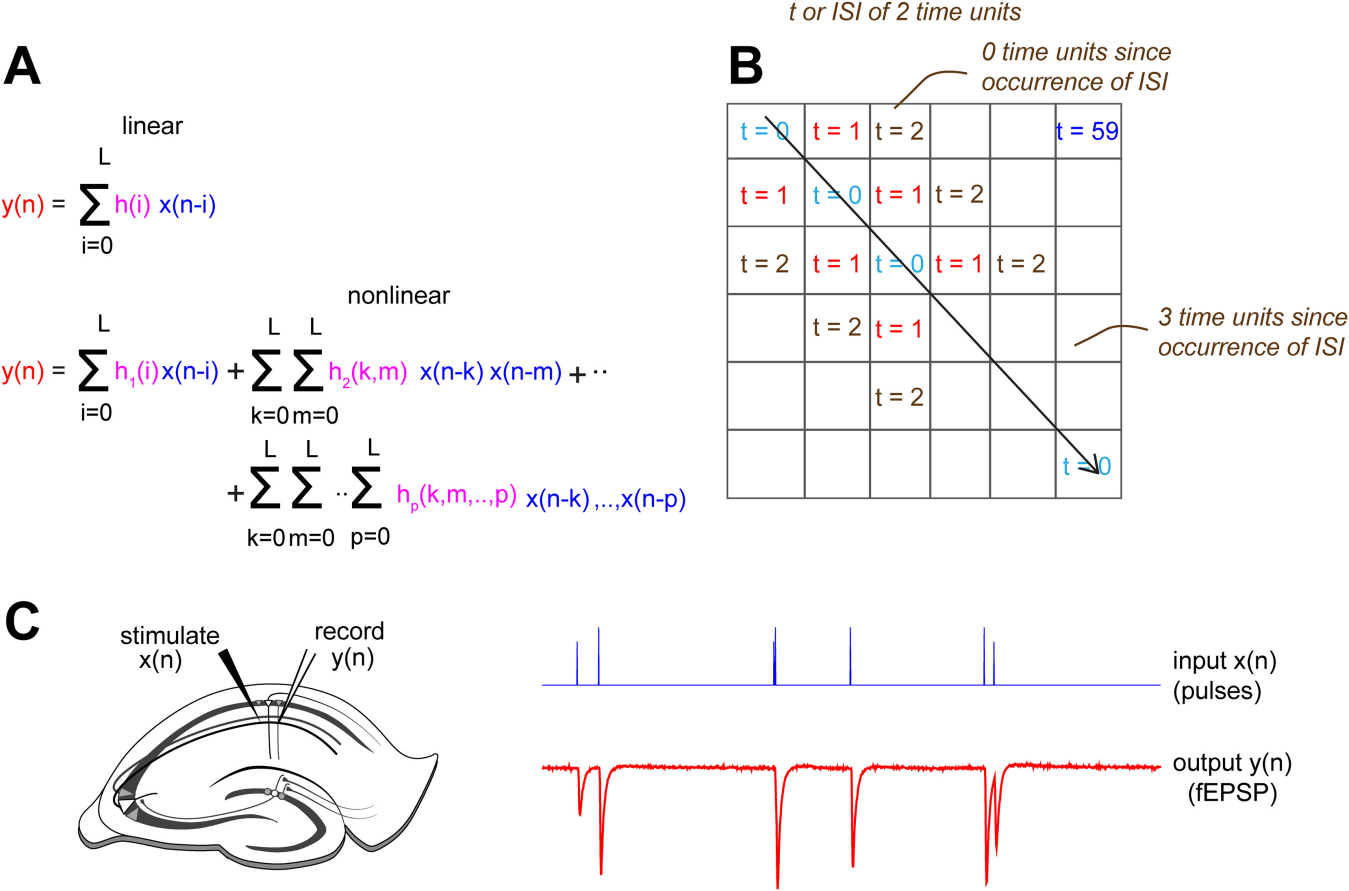
Using Volterra series expansion (VSE) to identify the CA1 apical dendritic operations. **(A)** Corresponding methods to fully characterize linear and non-linear systems. *h(i)* fully characterizes a time-invariant linear system, and *h(i)* up to *h_*p*_ (k,m,.,p)* fully characterize a non-linear system with p^th^-order non-linearities. **(B)** *h(k,m)* is a 2-d symmetric matrix of weights. *h(k,m)* is a function of two parameters, the inter-spike-interval (ISI, or τ) represented by a fixed diagonal, and how far in the past the ISI occurred (moving along the diagonal). **(C)** Schematic of slice electrophysiology stimulation and recording setup for the apical dendrites. Inputs *x(n)* were delivered to the Schaffer collaterals and outputs *y(n)* were recorded from the CA1 apical dendrites.

Characterizing a system’s operations (i.e., identifying its transfer function) differs depending on whether the system is linear or non-linear (note: here we extend the use of the term “transfer function” to apply to both linear and non-linear systems). For a linear, time-invariant system, estimating the impulse response function *h(i)* would fully characterize the system, but a non-linear system requires higher order terms (h(k,m),., h(k,m,.,p), Figure 1A). We identified, up to the second order, the CA1 apical dendritic system transfer function by estimating both the first and second order kernels *h(i)* and *h(k,m)*, respectively, to capture linear and non-linear dynamics, respectively, using the Volterra series expansion (VSE) for system identification (Marmarelis, 2004). This general form of the VSE fully captures n^th^ order system dynamics in an agnostic manner, with no prior assumptions about the system, and thereby avoids the generation of partially complete or misleading models (Marmarelis, 2004). Here, we have truncated the series expansion by making two assumptions: (1) system order, p, was set as 2 whereby only quadratic non-linearities of the system are captured and any higher order non-linearities that might be present are ignored, and (2) system memory, L, was set as 60 ms. These model parameters were selected *a priori* given the compromise between model complexity and practical considerations of computational and experimental burdens.

The term *h(i)*, the first order kernel, is a vector of weights on current and past input values that contribute to the current output. The term *h(k,m)*, the second-order kernel, is represented by a symmetric matrix of weights describing the relative influence of past pairs of inputs on a current fEPSP (Figure 1B). The values on its diagonal slices indicate how the product of two input values τ time points apart affect a subsequent response occurring after various delays. Values further along a given diagonal (a fixed interval between inputs) reflect weights for the multiplication of neighboring inputs that occurred even further in the past relative to the current output (i.e., the memory of the system, and more simply, the CA1 record of its input history, Figure 1B). A simplified schematic description for the pertinent variables of a second order Volterra series is illustrated in Supplementary Figure 1.

To estimate the first and second order kernels, we stimulated the Schaffer collaterals in young adult mouse hippocampal slices with pulse train *x(n)* and recorded the output signals *y(n)* from the proximal apical dendrites of the CA1 (Figure 1C). For each slice (10 slices, *n* = 20 sessions), input signals were delivered in two 15-min sessions. Stimulation pulse timing was drawn from a Poisson distribution with a pulse rate of 2/sec and with amplitudes chosen uniformly from two values: *x*_1_ which induced a sub-maximal fEPSP response *y*_1_ of around 3–4 mV, and *x*_2_ as a fraction of *x*_1_ (either 1/2 or 3/4). These input properties and experiment durations were carefully chosen through simulations to ensure kernel estimation accuracy (Supplementary Figure 2).

### CA1 apical dendritic kernel estimates

**FIGURE 2:**
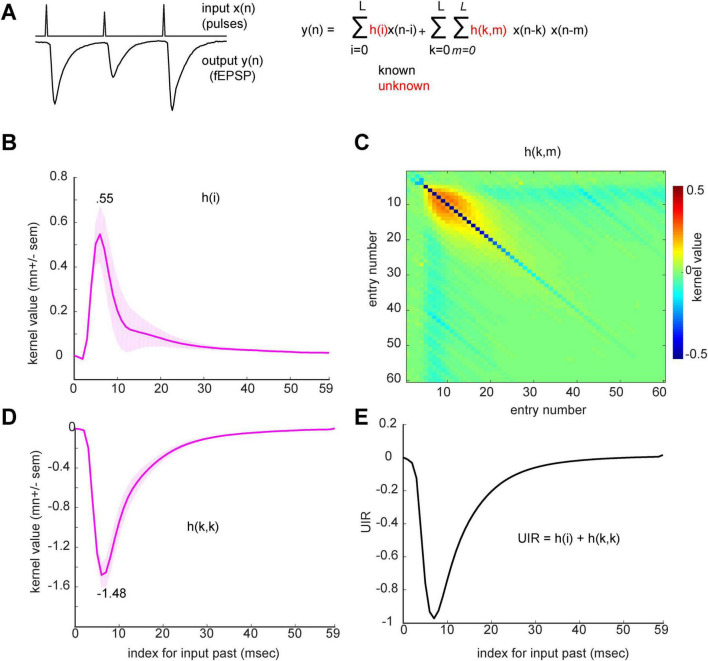
CA1 apical dendritic kernel estimates. **(A)** Example input stimulation and output CA1 apical dendritic recording data (left) and second order truncated Volterra series expansion (right). **(B,C)** The identified CA1 apical dendritic system; *h(i)* **(B)**, *h(k,m)* **(C)**. **(D)** *h(k,m)* main diagonal slice, *h(k,k)*. **(E)** system unit impulse response (UIR). Standard error shades reflect standard error of the mean (SEM) across sessions.

Estimation of kernels is reformulated as a least-squares regression problem (see Methods in Supplementary material). Group kernel estimates were obtained using the last 12 min of each 15-min session from each animal and after normalizing the input-output data. The linear (1st order) kernel is an estimate of the filtering properties and reflects how the current and past input values are weighted to contribute to the generation of the fEPSP. Such linear weights reached a maximum in 10–15 ms and then exponentially decayed toward zero during the following 50 ms (Figure 2B). The 2nd order kernel showed that maximum non-linear influence on a current response also occurred when past pulses were temporally close to each other (Figure 2C). This is evident in the matrix plot, from the higher magnitude kernel values in slices immediately off the main diagonal. Second order main diagonal entries reflect the weights for the contribution of the squared values of the input in generating the response (Figure 2D). The unit impulse response, which is the sum of the first order kernel and second order main diagonal entries, is by definition a system’s expected response to a delta-function (i.e., a stimulation pulse), which in the present case is the fEPSP (Figure 2E). The result matched the complete waveform of a typical fEPSP recorded in the proximal apical dendrites (Figure 2A). Note: individual animal *h(i)*, *h(k,m)* and *h(k,k)* estimates are included in Supplementary Figures 13–15, respectively.

### Derived kernels generalize across animals with high prediction accuracy

**FIGURE 3:**
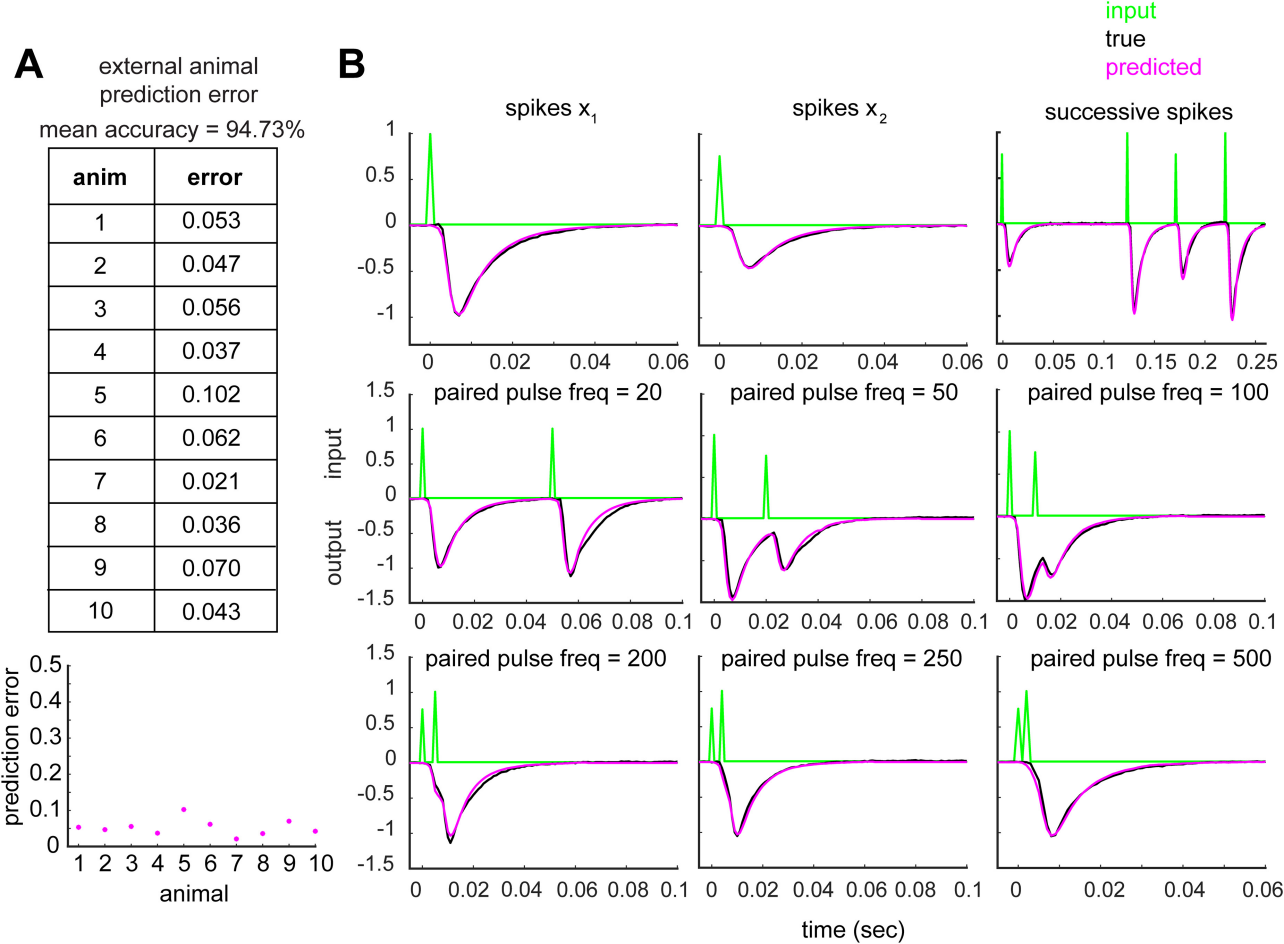
Estimated CA1 transfer function reliably predicts CA1 output in new animals. **(A)** Table (top) and plot (bottom) displaying external animal prediction error (model generated using n-1 animals, with the test animal excluded). **(B)** Example of predictions from animal 8 data for isolated spikes (top row, first two panels), successive spikes (top row, third panel), and paired pulses of variable ISIs (remaining). All examples were extracted from a continuous 15-min recording.

We then tested if the transfer function accurately reproduces outputs from slices from different animals that were not included in the original derivation. To do so, data from 9 animals were used to estimate the transfer function, which was then used to predict the CA1 output for the 10th animal (Leave-one-out (LOO) cross validation, Figure 3A). Normalized external prediction accuracy (normalization was relative to the magnitude of the true output; detailed in Methods in Supplementary material) across the 10 animals was 94.73%. The transfer function was therefore generalizable to new data, and reliably described the output across complex input patterns (Figure 3B).

### The non-linear component of the apical dendritic operations outweighs the linear one in generating the dendritic response

**FIGURE 4:**
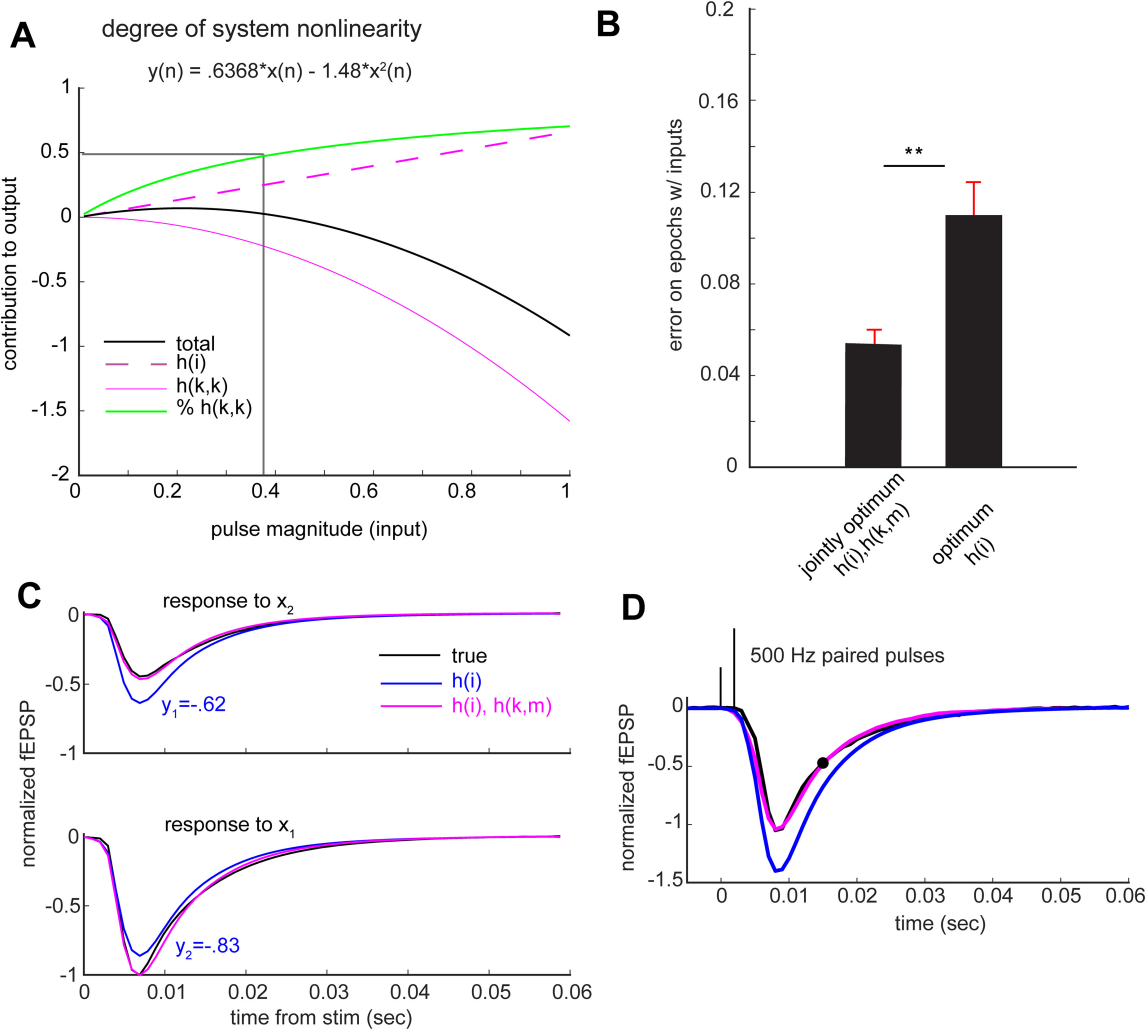
The non-linear component of the apical dendritic operations outweighs the linear one in generating the dendritic response. **(A)** Quantification of system non-linearity. Using the peak values of the *h(i)* and *h(k,k)* (indicated in Figures 2B, D), the predicted system output peak value (black) is plotted as a function of the input. Here, system output (black) is defined as a univariate value (the peak value of the fEPSP) rather than the continuous fEPSP waveform. The contributions of *h(i)* and *h(k,m)* to the output are displayed in magenta dashed and solid lines, respectively. The fraction of the output generated by the non-linear system component is shown in green. The intersection of the vertical and horizontal lines indicates that the non-linear contribution to the output outweighs the linear one for input ranges larger than ∼0.4. **(B)** Differences in left-out animal prediction error using jointly optimum *h(i)*, *h(k,m)* and the optimum *h(i)*, reflecting non-linear and linear systems, respectively (*p* < 0.001, paired non-parametric permutation testing). **(C)** Black traces reflect mean true response to x_1_ (bottom) and x2 (top) to isolated spike inputs (not preceded nor followed by spikes in the 1-sec and 0.25-sec periods relative to stimulation, respectively). Predicted waveforms by linear and non-linear CA1 models are shown in blue and magenta, respectively. Linear CA1 model applies superposition (y2 is the same linear combination of y1 as x2 is of x1; y2 = 3/4 × y1). **(D)** An input of two spikes that are 2 ms apart is shown in black. Part of the predicted output value at 15 ms (black circle) is generated by the non-linear term whereby the product of the two input pulses is weighted by an entry of the matrix slice that is 2-off the diagonal.

The above findings raise questions about the relative contributions of the linear vs. non-linear components of the equation to the fEPSP waveform. The magnitude of the 2nd order kernel diagonal values (1.48) was larger than that of the 1st order kernel (0.55, see Figures 2B, D, above). Using these two values, the contribution of the linear component (*h(i)*) and non-linear component (*h(k,m))* to the peak output can be calculated as a function of input strength (number of afferent axons stimulated). The ratio of non-linear-to-linear contribution to the response increases with increasing magnitudes of the input (the square of the input is larger, thereby applying *h(k,m)* weights to this larger square contributes more greatly to the output). For the CA1 apical dendritic system, the non-linear component outweighs the linear one in generating the output at input values larger than ∼0.4 (translates to 40% of the input that induces a submaximal response, Figure 4A); see Supplementary Figure 19 for individual animal estimates.

The above results indicate that the non-linear component of the transfer function is essential for generating the dendritic output. We quantified this by measuring the drop in output waveform prediction accuracy when using only a first order kernel compared to using both the first and second order kernels. To do so, we estimated two transfer functions; an optimum stand-alone *h(i)* kernel and jointly optimum *h(i)* and *h(k,m)* kernels. We found that prediction error on left-out data was significantly lower when using the transfer function of a non-linear CA1 system in contrast to a purely linear system (*p* < 0.001, paired non-parametric permutation testing) (Figure 4B).

The above analyses included multiple instances in which a single stimulation pulse was not preceded by another pulse for 1000 ms. Examination of these cases led to an informative result: an estimated linear system does not accurately predict changes in fEPSP amplitude associated with changes in single pulse stimulation current (Figure 4C). Thus, non-linearity is at least partly introduced by shifts in the number of active afferent fibers. Non-linearity is also differentially recruited by differential spike timing; if a system was linear in spike patterns, then h(k,m) would have been a zero matrix (not observed in Figure 2C), and h(i) would have been sufficient to predict the output to a given input spike pattern.

This is of interest given an existing notion that the CA1 system, in contrast to other hippocampal subdivisions, operates in a linear manner (Marmarelis, 2004; Schulz et al., 2018) (see section “Discussion”) (Knierim and Neunuebel, 2016; Yassa and Stark, 2011). The combination of different input sizes arriving in the recent past, in addition to their variable timing (pattern) which influences how the dendrites respond (*h(k,m)*), together lead to further error accumulation and thus further reduction in the predictive power of the 1st order kernel with regard to the fEPSP waveform (Figure 4D for an example). Including the estimated non-linear transfer function allows for tracking of isolated inputs of variable magnitudes as well as arbitrary pulse patterns by accounting for the lag between all possible pairs of inputs and the time of occurrence of such lags.

### New timing rules in the apical dendrites

**FIGURE 5:**
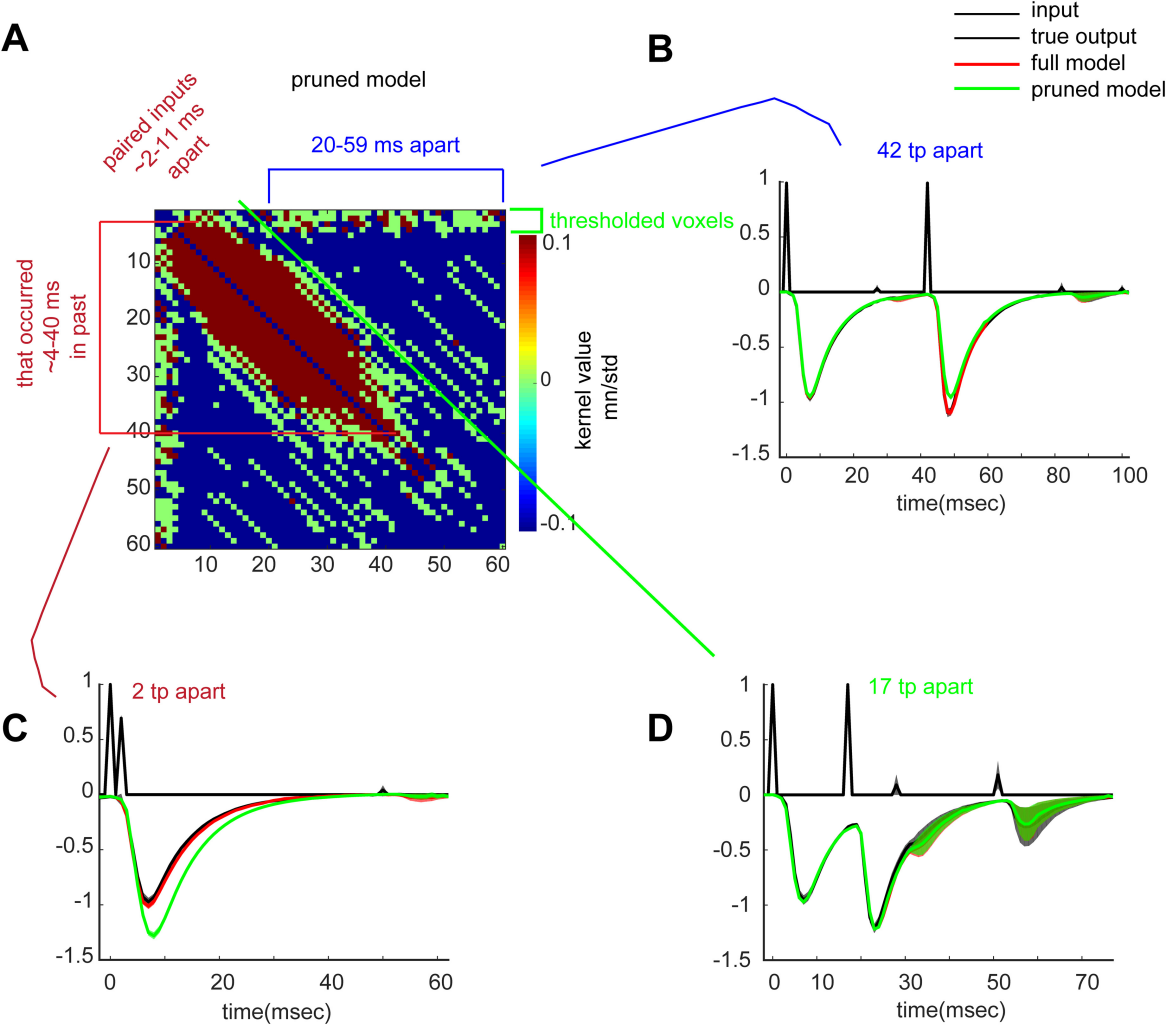
New timing rules in the apical dendrites. **(A)** Pruned *h(k,m)* displays a thresholded matrix where included pixels are those in the top (red) and bottom (blue) 20th and 40th percentiles, respectively, of the standardized mean (mean/standard deviation) kernel value distribution. Removed entries are indicated in green (such entries are close to 0 and thus the system does not employ them for output generation). Positive weights (red bracket) are applied for input pairs with an ISIs of 2–11 ms. Negative weights are present for ISIs of 20–59 ms (blue pixels). **(B)** Display of the importance of negative weights. Another pruned model was generated that excluded negative weights. Shown are full model predictions (with negative weights, including those for ISI of 42; red trace), pruned model (excludes negative weights, including those for ISI of 42; green trace), and true input and output (black trace). Negative weights are needed for an accurate prediction. **(C)** same as in panel **(B)** but for positive weights. Positive weights are needed for an accurate prediction. **(D)** same as in panel **(B)** but for green (thresholded) weights. Green pixels are not necessary for an accurate prediction.

The matrix of second order kernel weights provides a means for identifying essentially any 2nd order non-linear interactions between past inputs and present responses, many of which would go undetected in conventional physiological studies. To facilitate this application, we developed a pruned second order kernel which included values that were either below or above the 60th and 80th percentiles, respectively, of the mean divided by the standard deviation of entries across all sessions (consistently large negative and positive weights, respectively, across sessions) (Figure 5A). As a general point, the matrix shows that consecutive pulses occurring in rapid succession (∼2–11 ms) in the recent past (∼4–40 ms) will depress current responses (less negative fEPSP; red pixels) while twin pulses separated by longer intervals (∼20–59 ms) will exert a facilitative effect starting at 10 ms onward after delivery of the second pulse (blue pixels). Importantly, the initial segment of the slope of the second pulse (∼1–10 ms) is largely unaffected by non-linear components of the dendrites (green pixels). Given the high accuracy of predictions from the transfer function, we can be confident that these physiological rules describe outcomes when, as *in vivo*, target cells receive continuous input. We tested for the presence of these rules in the recordings, and they were indeed present (Figures 5B–D).

### Signal transformation in CA1 basal dendrites

#### A transfer function for the CA3 projections to CA1 basal dendrites

**FIGURE 6:**
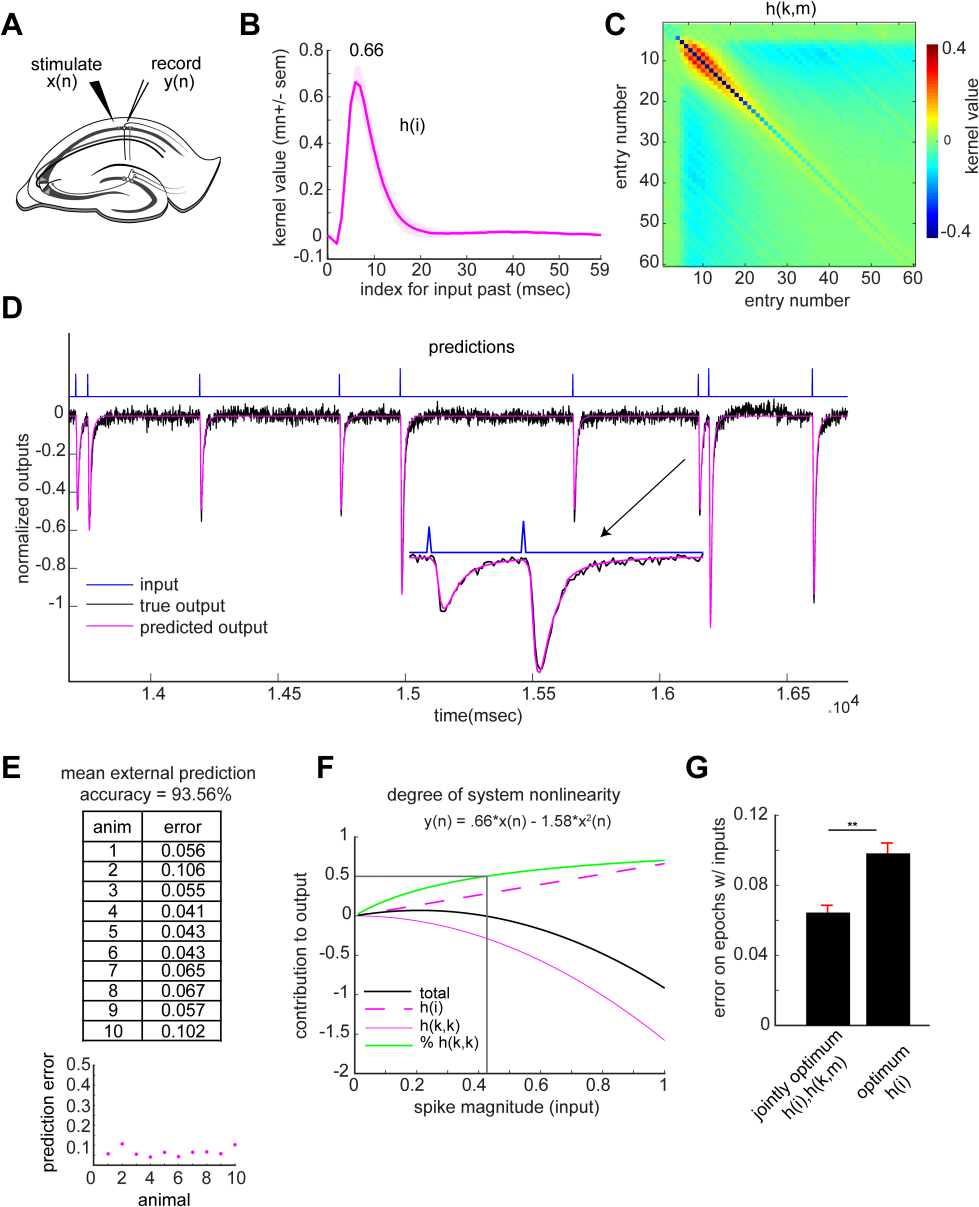
Signal transformation in the basal dendrites of CA1. **(A)** Schematic of slice electrophysiology stimulation and recording setup for the basal dendrites. Inputs *x(n)* were delivered to the Schaffer collaterals and outputs *y(n)* were recorded from the CA1 basal dendrites. **(B,C)** The identified CA1 basal dendritic system; *h(i)* **(B)**, *h(k,m)* **(C)**. **(D)** Example trace from one recording session displays inputs (blue), true induced output (black) and externally predicted output (magenta). Inset shows a larger display of the paired spike segment of the trace. **(E)** Table (top) and plot (bottom) display external animal prediction error (model generated using n-1 animals, with the test animal excluded). **(F)** Quantification of system non-linearity. Using the peak values of the *h(i)* (indicated in B) and *h(k,k)* (indicated in Supplementary Figure 3A), the predicted output peak value (black) is plotted as a function of the input. The contributions of *h(i)* and *h(k,m)* to the output are displayed in magenta dashed and solid lines, respectively. The fraction of the output generated by the non-linear system component is shown in green. The intersection of the vertical and horizontal lines indicates that the non-linear contribution to the output outweighs the linear one for input ranges larger than ∼0.41. **(G)** Differences in external animal prediction error using jointly optimum *h(i)*, *h(k,m)* and the optimum *h(i)*, reflecting non-linear and linear systems, respectively (*p* < 0.01, paired non-parametric permutation testing).

Little is known about the dynamics of synaptic transmission in the CA1 basal dendrites. We accordingly employed the same system identification procedures used in the analysis of the apical dendrites to derive a transfer function for the basal domain. Stimulation pulses were delivered to a site in the distal stratum oriens of field CA1c (proximal CA1) approximately 400 μm removed from a recording pipette located at the same level of the lamina (Figure 6A). As above, stimulation pulse timing was drawn from a Poisson distribution with mean spike rate of 2 Hz and pulse amplitude was drawn uniformly from two values x_1_ and a fraction of x_1_ (*n* = 10 slices, *n* = 37 sessions; Supplementary Table 2). The 2nd order VSE was estimated as described in the earlier section on the apical dendrites. The obtained solution involved a sharp upward going first-order kernel *h(i)* which reflects system linear dynamics and an *h(k,m)* matrix which reflects system 2nd order non-linearities (Figures 6B, C). The main diagonal slice from the matrix produced downward going weights over time, reflecting the weights for the contribution of the squared values of the input in generating the response (Supplementary Figure 3A). The unit impulse response – which is the system’s response to a single pulse – again matched the mean waveform of the basal fEPSP (Supplementary Figure 3B). Individual animal *h(i)*, *h(k,m)* and *h(k,k)* estimates are included in Supplementary Figures 16–18, respectively.

#### Derived kernels generalize across animals with high prediction accuracy

The derived transfer function reproduced fEPSPs across various stimulation patterns (Figure 6D). To quantitatively evaluate accuracy, the basal dendritic transfer function was estimated using data from n-1 animals and then used to predict the dendritic response on the n^th^ animal (LOO). External prediction was done for all sessions and slices with the results summarized for each animal (Figure 6E). The solution is shared from animal to animal, predicting the entirety of the synaptic response on a msec-by-msec basis with a mean external animal prediction accuracy of 93.56%. Calculations of the relative contributions of the linear and non-linear elements yield a result similar to that for the apical dendrites: the non-linear contribution outweighed the linear one as stimulation pulse intensity increased (Figure 6F); see Supplementary Figure 19 for individual animal estimates. As expected, the external prediction error significantly increases when estimating only an optimum *h(i)* solution to represent the optimum linear CA1 basal dendritic system (Figure 6G)—evidence for the necessity of the non-linear operations for reconstructing the dendritic response.

#### New signal transformation rules in the basal dendrites

**FIGURE 7:**
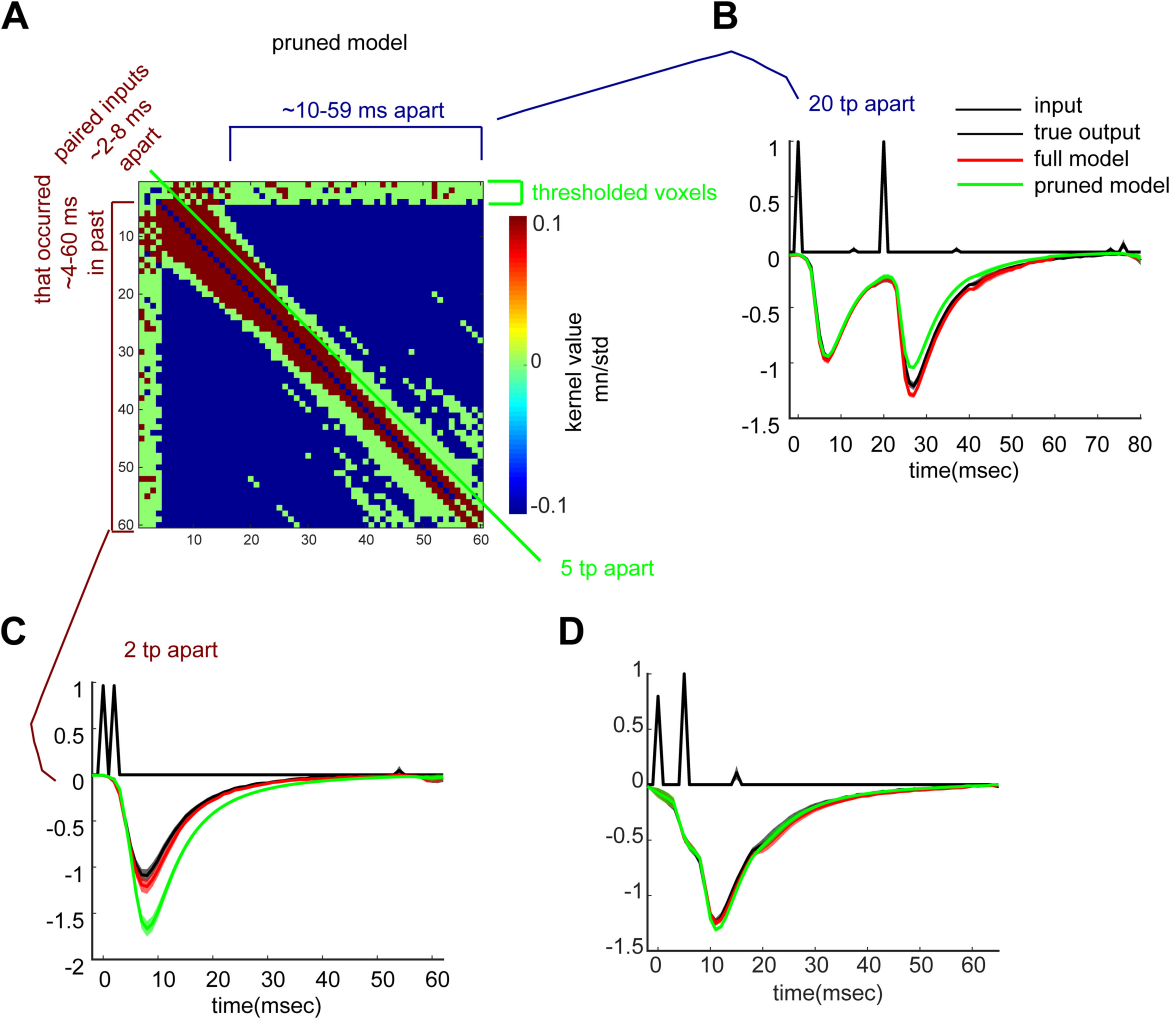
New timing rules in the basal dendrites. **(A)** Pruned *h(k,m)* displays a thresholded matrix where entries in the top (red) and bottom (blue) 13th and 65th percentiles, respectively, of the standardized mean (mean/standard deviation) kernel value distribution were included. Removed entries are indicated in green (such entries are close to 0 and thus the system does not employ them for output generation). Positive weights (red bracket) are applied for input pairs with an ISI of 2–8 ms. Negative weights are present for ISIs of 10–59 ms (blue pixels). **(B)** Display of the importance of negative weights. Another pruned model was generated, whereby negative weights were excluded. Shown are full model predictions (includes negative weights, including those for the ISI of 20; red trace), pruned model (excludes negative weights, including those for the ISI of 20; green trace), and true input and output (black trace). Negative weights are needed for an accurate prediction. **(C)** same as in panel **(B)** but for positive weights. Positive weights are needed for an accurate prediction. **(D)** same as in panel **(B)** but for green (thresholded) weights. Green pixels are not necessary for an accurate prediction.

A pruned version of the 2nd order kernel was again obtained by including only entries whose mean divided by the standard deviation across slices was lower or higher than the 65th and 87th percentiles, respectively (Figure 7A). With satisfactory agreement to the recorded data, the pruned matrix shows which input patterns generate more positive (red pixels) versus negative (blue pixels) fEPSPs by second order non-linear dynamics, and that again, the slope response to the second pulse is largely unaffected by the non-linear dynamics (green pixels) (Figures 7B–D for examples).

While the basal dendrites’ *h(k,m)* revealed non-linear dynamics that were indeed present in the data, in examining paired pulse prediction errors, we discovered that *h(k,m)* predictions are inaccurate for some cases of paired pulses (Supplementary Figures 4–7). However, paired predictions held for most cases in the apical dendrites (Supplementary Figures 8–11). These observations indicate that the apical dendrites are sufficiently explained by 2nd order dynamics, while the basal dendrites require a 3rd order kernel or higher for complete characterization (Sclabassi et al., 1988).

### Comparison of signal transformation in the apical vs. basal dendrites

#### Signal transformations in the apical differ from those in the basal dendrites

**FIGURE 8:**
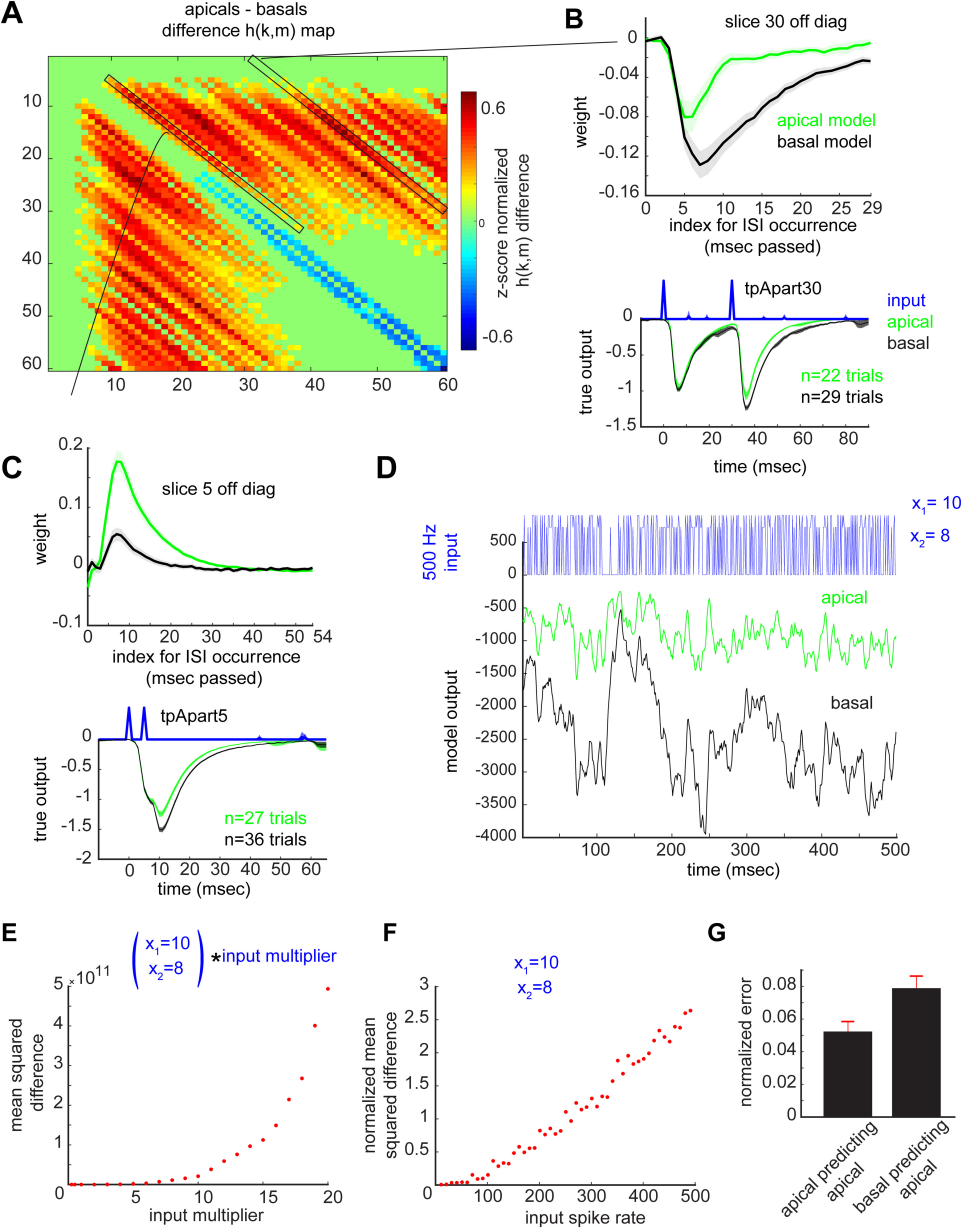
The apical transfer function cannot generate basal output signals and vice versa. **(A)** z-score normalized apicals-basals difference *h(k,m)* map, cluster corrected for multiple comparisons (CBPT, *p* < 0.05, using null distribution cluster size to correct for multiple comparisons, and using sessions as observations). Green pixels are *h(k,m)* values that did not significantly differ between the two regions. Warm and cool pixels are significantly higher and lower *h(k,m)* values for the apicals compared to the basals, respectively. **(B,C**, top**)** A group mean trace of kernel values across sessions for a given off diagonal slice in the matrix (slice 30 = 30 ms ISI **(B**, top**)** and slice 5 = 5 ms ISI **(C**, top**)** off the diagonal), indicated with black rectangles in A, for the apical (green) and basal (black) models. **(B,C**, bottom**)**, Mean input and output traces of all spikes with 30 ms (B, bottom) and 5 ms (C, bottom) ISIs pooled from all apical (green) and basal (black) recording sessions. Note the relatively less negative apical decay phase is consistent with the identified non-linear system operations **(B,C**, top**)** whereby the addition of more largely weighted paired input products to the remaining terms in the VES yields a less negative output. **(D)** A 500 ms segment of the apical and basal transfer function output to a Poisson input with mean rate of 500 Hz and spike magnitude uniformly drawn from x_1_ = 10 and x_2_ = 8. **(E,F)** Mean squared difference between apical and basal transfer function outputs as a function of input magnitude **(E)** and input spike rate in Hz **(F)**, respectively. **(G)** External prediction error on pooled high amplitude successive spikes x_1_ and the 60 ms period following the second spike, for all spikes with ISIs of 1–60 ms apart. Basal cross prediction error was significantly higher when predicting the output waveform in the apical dendrites (*p* = 0.01, unpaired permutation testing, *n* = 100 permutations). Error shades and bars represent SEM across sessions.

There were general similarities between the two dendritic domains; no significant differences were found between the basal and apical *h(i)* and *h(k,k)* (Supplementary Figures 3C, D. Direct comparisons of *h(k,m)*, however, revealed significant differences in the two dendritic domains. For successive stimulation pulses whose ISI was 2–3 ms, the apical dendrites apply smaller weights on the fEPSP thus yielding a more negative fEPSP compared to the basal dendrites (blue pixels, Figure 8A). The converse is true for a larger range of ISIs, anywhere from ∼3–59 ms ISIs, whereby the apical dendrites apply larger weights, thereby generating less negative fEPSP decays compared to the basals (red pixels, Figure 8A). To examine this further, diagonal slices of *h(k,m)* reflecting the weights for the input history of paired pulses arriving with an ISI of 30 ms apart were plotted separately (Figure 8B, top). Such traces clearly revealed that compared to their basal counterparts, apical dendrites apply less negative weights (less facilitation) generating a less negative output ∼7–29 ms after the occurrence of the second pulse (after the occurrence of the ISI event).

We next confirmed the presence of such dynamics in the experimental data, using group mean outputs induced by ISIs of 30 ms (Figure 8B, bottom). Indeed, the decay phase of the apical response to the second pulse, separated from the first by 30 ms, is less negative compared to that of the basals. Another example is shown for the *h(k,m)* slice reflecting weights for paired pulses with an ISI of 5 ms (Figure 8C, top). The larger positive weights in the apicals compared to the basals should yield, again, a less negative fEPSP ∼5–35 ms after the occurrence of the second pulse, which is indeed observed in the group average of such trial types (Figure 8C, bottom). In addition to the significant differences identified in the wings of *h(k,m)*, it is also important to note that the magnitude of the raw (non-normalized) fEPSP differed between the domains; the basal domain generates a more compressed version than that of the apical domain (Supplementary Figure 3E). This suggests that the two domains’ *h(i)* and *h(k,k)* are comparably similar in waveform, but are rescaled versions of each other, while *h(k,m)* for *k*≠*m* differs significantly (Supplementary Figure 3F).

#### The apical transfer function cannot generate basal output signals and vice versa

Two characteristics of our data mask the extent to which the two dendritic domains are capable of generating different analog output waveforms. The first of these is the output magnitude, which is capped at ∼1 due to the normalization. Yet, the contribution of the system non-linearities to the output for both the apicals (Figure 4A) and basals (Figure 6F) increases with input magnitude, and it is in the non-linear operations that the two systems differ. Secondly, our experimental protocol relied on a slow mean spike rate (2 Hz), meaning that on average, pairs of spikes were 500 ms apart. However, the weights in *h(k,m)* which significantly differed between the two systems cover a range of ISIs of 1–59 ms which translate to a spike frequency range of 17–1000 Hz. Therefore, our experimental protocol of small magnitude inputs and slow spike rates does not produce inputs that tap directly into the difference in dynamics between the two systems. We therefore took advantage of our discovery of such differences together with accurate transfer function estimates and examined the basal and apical model behaviors to inputs with increasing magnitudes and spike rates (Figure 8D). Basal and apical model outputs are very different with high input magnitudes and spike rates. Quantifying the mean squared difference between the two systems’ responses to the same input shows that the magnitude of the difference increases with increasing input magnitude (Figure 8E) and spike rate (Figure 8F).

Lastly, as significant differences between the apical and basal dendritic transfer functions were observed at the level of the second order kernel *h(k,m)*, which reflects system operations on pairs of pulses, we tested cross-transfer function predictions on such successive pulses. We found a significant increase in external prediction error on pooled epochs with successive pulses (1–60 ms apart) from all animals, when using the basal transfer function to predict the output waveform to arbitrary inputs delivered in the apical dendrites (Figure 8G).

### Identifying sources of prediction error

Several sources of prediction error were identified. First, CA1 dendritic system memory is longer than the 60 ms interval considered in the study. One way to demonstrate this is through the paired pulse analyses (Supplementary Figures 4–11), where predicted traces deviate from true CA1 responses starting with the 60th msec timepoint after the first pulse. After the 60th msec, the predicted output response is generated by operations on the second pulse alone (the first pulse falls outside of the model’s 60 msec memory) while the CA1 response is still responding to both pulses (retained memory for the first pulse that occurred over 60 ms in the past).

Non-stationarity in the data is another source of prediction error (Supplementary Figure 12). While the majority of recordings appeared to be stationary (showed a constant induced response magnitude as a function of the recording duration, Supplementary Figure 12A), a subset was non-stationary (i.e., a gradual change in the magnitude, see Supplementary Figure 12D). Volterra kernels are only applicable for modeling stationary systems, as the kernels are assumed to be time invariant. They are estimated by minimizing the squared-error difference between the actual and predicted model outputs; therefore, for non-stationary data, the kernels will have higher prediction accuracy in the subset of the recording that is most similar in range to that of the group level induced CA1 responses (Supplementary Figure 12F).

A third source of error (more relevant for the apical model) was the variable *h(i)* across animals (see SEM shades in the grand average Figure 2B and individual animal estimates Supplementary Figure 13). Variability in *h(i)* was visible in terms of both waveform shape and magnitude. This difference in *h(i)* is theoretically consequential and informs as to the nature of system output as a function of input (Supplementary Figures 19A, B). For example, the *h(i)* estimate for apical animal 5 varied compared to estimates for other animals (Supplementary Figure 13). The magnitude of this *h(i)* coupled with animal 5’s *h(k,k)* estimate will dictate the linear and non-linear contributions to the system output (as discussed for Figure 4A). Because *h(i)* is large in magnitude, it will predominate in generating the output over a larger range of the normalized input (rightward shift in Supplementary Figure 19C). In addition, due to the fact that it is an upward going waveform, it will generate a less negative fEPSP for such input ranges. These properties were unique to this animal, hence the different input-output curve (Supplementary Figure 19A), and the highest external animal prediction error (Figure 3A).

Lastly, examination of paired pulse prediction error suggests that a model with higher order than 2 is needed for the basal domain (this was clearly evident in Supplementary Figure 7, plots boxed in black).

## Discussion

The primary objective of the present work is to develop a systematic and concise mathematical characterization of signal transformation for inputs arriving from field CA3 and terminating in the dendrites of field CA1. This entailed analysis not only of inputs of different strength but more importantly the influences exerted by prior and current inputs on present responses. The endpoint measure was the generation of the entirety of the fEPSP which is a direct reflection of the excitatory post-synaptic current (EPSC)—the primary unit of communication between cortical regions. The system developed here predicted the size and waveform of the dendritic response, on a msec-by-msec basis, with a high degree of accuracy.

While our results show that the model generalizes across animals and spike patterns the ultimate goal is a transfer function that generalizes to the *in vivo* setting, reliably reproducing CA1 outputs to CA3 *in vivo* inputs. In the slice set up utilized here, axonal inputs and therefore modulations from other brain structures were eliminated. However, we utilized a CA3 input spike distribution that closely resembles that of the *in vivo* setting - in other words, a CA3 input distribution that is a likely outcome of the variable and dynamic neuromodulatory states from differentially recruited distant and local brain areas (see Methods in Supplementary material). An advantage of having a random spike input pattern from a distribution that approximates *in vivo* CA3 inputs was to cover a large sample space of possible inputs, which may cover the diverse *in vivo* CA3 spike patterns that result from different neuromodulatory states. One of the reasons we opted not to begin our investigations in the *in vivo* setting using naturally generated CA3 inputs is because such input space would be limited to the duration of the recording and the exact neuromodulatory state of context/behavior; this would represent only special cases of inputs. However, explicit testing is needed to determine whether these transfer functions reliably generate *in vivo* CA1 outputs to concurrently recorded CA3 *in vivo* inputs.

For the present model, further reductions in prediction error will likely be obtained by extending the basal dendritic system order beyond 2nd order possibly to account for active dendritic components that exhibit greater than 2nd order non-linearities which cannot be captured by the present model and dendritic system memory beyond 60 ms in accord with experimental results (Magee and Johnston, 2005; Ness et al., 2016; Sinha and Narayanan, 2022). Furthermore, extending system memory could minimize trial to trial variability, by better accounting for input spike pattern content in the more distant past. Lastly, biological variability from animal to animal as demonstrated in our results could contribute to the model’s error.

We found that deviations of CA1 system dynamics from linearity can be quantified in a generalizable manner and are dictated by the magnitude of the ratio of *h(k,m)* to *h(i)*. The larger the ratio, the earlier the deviation occurs at smaller values of the input. Non-linearities made up more than 50% of output magnitude at normalized input values larger than ∼0.4 (translates to 40% of the input which induces a submaximal response); at these values, the second-order kernel contributes more to the fEPSP waveform. There is sufficient experimental and modeling evidence that dendritic morphology and expression lends to both linear (passive current flow across the membrane) and non-linear (active conductance) processing (Ness et al., 2016; Sinha and Narayanan, 2022). Such non-linear dendritic processing extends to the CA1 dendrites; components of the CA1 dendritic structure that give rise to non-linear processing include v-gated Na+ and K+ channels (Magee and Johnston, 2005), GABAergic interneurons (Tzilivaki et al., 2022), and dendritic spikes and on the relevant dendritic branch morphologies (Losonczy and Magee, 2006). Despite this, there remain discussions of the CA1 being a linear system when contrasted with other hippocampal subdivisions with implications that the CA1 is not the underlying structure mediating mnemonic pattern separation nor completion but rather transmits a 1:1 input-output read out after such computations ensue in other hippocampal fields (Amani et al., 2021; Guzowski et al., 2004). Our results here are consistent with the former set of prior evidence for the existence of non-linear dendritic dynamics. However, they do not accord with contemporary arguments that posit marked differences in non-linear input/output relationships for the dentate gyrus and field CA3 with CA1 having a more linear relationship (Knierim and Neunuebel, 2016; Yassa and Stark, 2011). One effect of these proposed subfield differences is to allow for pattern separation by the divergent afferents of the dentate gyrus and pattern completion by the convergent interior of CA3 with CA1 providing reliable readout. There is empirical evidence for this general model, but our results suggest that adjustments are required for its output stage (Guzowski et al., 2004; Knierim and Neunuebel, 2016; Yassa and Stark, 2011).

Field EPSPs are commonly used to evaluate the effects of experimental treatments such as drugs or genetic mutations on brain operations. Studies of this type typically employ widely spaced stimulation pulses and single measures of the evoked response such as slope or amplitude to test for changes from baseline; they only rarely include information about the dynamic properties of synapses as elicited by temporal patterns of inputs. The comprehensive and agnostic approach followed in the present study has led to a readily implemented means for obtaining a far more complete description of synaptic operations. The *h(i*) and *h(k,m)* terms fully characterize the behavior of the 2nd order apical system with a given memory and allow for a simple but accurate measurement of the distance in synaptic operations from a baseline state to that produced by a condition of interest. Such conditions include experimental manipulations like varied genetics, pharmacological agents, sex, age, and importantly, inter-subject heterogeneity. Regarding the latter, this approach can provide a means to quantify where synaptic operations may be preserved across individuals and where they may vary, with variation potentially being ascribed to individuality (likely partly an accumulation of life experiences). Therefore, an accurate model may be bounded in its cross-prediction accuracy because there is likely model specificity to each brain. These features should result in a sizable gain in sensitivity and thus greater discrimination between the changes elicited by experimental manipulations.

Similar arguments apply to ongoing attempts to comprehensively describe the differences in signal processing by the various links in the hippocampal circuitry. Multiple studies have shown that the pertinent synapses respond in surprisingly different ways to repetitive stimulation delivered at frequencies corresponding to dominant EEG rhythms. For example, the apical branch of the CA3 to CA1 projections exhibits a conventional frequency facilitation effect to 5, 20, and 50 Hz trains (Jackman et al., 2016) while the lateral perforant path connections to the dentate gyrus exhibit a form of low pass filtering (Amani et al., 2021). These results are informative regarding differences in underlying neurobiological mechanisms but constitute special cases that do not lend themselves to the extraction of general rules of the type discussed above. We accordingly established a transfer function for the input to the basal dendrites and found it to be clearly different than that for the apical tree. The majority of the h(k,m) entries significantly differed between the two regions (Figure 8A). The magnitude of the difference is even more apparent when plotting vectors from this matrix separately (Figures 8B, C). These differences are consequential in that for the same input, operations by respective estimated kernels generate vastly different outputs (Figure 8D). And, on the same fronts, using the basal kernels to predict the apical recorded output leads to a significantly higher prediction error (Figure 8G). The values for *h(k,m)* in the two domains differed because of evident discrepancies in the forward contributions of past events.

Moreover, a limited number of cases in which the basal dendritic transfer function did not fully predict the effects of closely spaced pulses strongly suggest that a 3rd or higher order kernel will be needed to fully characterize responses in this lamina. While techniques exist for estimation of larger order models while reducing the need for unrealistic amounts of experimental data, it is unknown *a priori* whether the functionals used in these approaches can represent the underlying ground truth Volterra kernels (see for example a discussion on the Laguerre Expansion Technique (Marmarelis, 2004)). Clearly, a combination of modeling and experimental work will be needed to develop a conceptually robust, higher order description for the basal dendrites.

There are several possible contributors to the differences in signal processing by the apical vs. basal dendrites. We observed a larger variability in individual animal *h(i)* estimates in the apical compared to the basal domain (see standard error shades Figure 2D vs. 2C). Since *h(i)* reflects the linear dynamics, that is for the dendritic system is largely passive conductance along the membrane, *h(i)* variability is likely a result of the more variable branching pattern of the apical dendritic membranes (Mihaljević et al., 2020). The small number of synapses activated by the weak stimulation pulses used in the present study are more likely to activate different electrotonic locations from experiment to experiment in the apical than basal dendrites. There were also differences between the two nodes in the non-linear aspects of the responses produced by multiple input pulses. For the dendritic system, non-linearities are activated as thresholds for components in the synapse are reached. Relevant to this, pyramidal cells express a large number of diverse potassium and calcium channels, certain of which are not uniformly distributed along dendritic branches (Ariav et al., 2003; Johnston et al., 2000; Migliore et al., 2018). The channels affect the size and waveform of synaptic potentials, are sensitive to recent input history, and in many cases operate over time frames comparable to those in our stimulation protocol. Accordingly, regional differences in channel types (Kirizs et al., 2014) could account for much of the observed discrepancy between the 2nd order kernels obtained for apical vs. basal domains. It is also the case that the two dendritic laminae contain different types of interneurons (Cossart et al., 2006; Klausberger and Somogyi, 2008; Schulz et al., 2018). Interneuron subtypes vary substantially with regard to their activation requirements and firing patterns; moreover, there is evidence for selective targeting of particular combinations of GABA_*A*_R subunits (Schulz et al., 2018). There is thus a possibility that feedforward shunting inhibition elicited by repetitive afferent stimulation will not be the same in the basal dendrites as in their apical counterparts. Consistent with this are results showing that the GABA-mediated depression of the later EPSPs in a short sequence evoked by closely spaced stimulation pulses is much more pronounced in the str. radiatum than str. oriens (Arai et al., 1994).

The pyramidal neuron with distinct dendritic domains is a defining feature of mammalian cortex. There has been considerable interest in the possibility that its multiple dendritic domains allow for processing of input from diverse sources. We extend to this in showing that processing differs in these two domains. Given the presence of two distinct sets of computations on the same cell suggests that the production of maximal depolarization, and thus likelihood of evoked spiking, requires input patterns that lead to a net larger depolarization generated by the joint transfer functions. It is of interest in this regard that tests for relationships between *in vivo* spiking patterns in CA3 and CA1 have utilized 3rd or higher order systems (Gholmieh et al., 2002; Song et al., 2007). In any event, the present findings are informative regarding hypotheses about the adaptive advantages of pyramidal cell architecture.

Finally, the findings reported here constitute a critical step in the development of hippocampal models from equations that incorporate a high degree of biological realism. Further progress in this direction will again require a transfer function for the conversion of EPSP sequences into spike patterns. Prior work using system identification techniques has established relationships between inputs and synchronous discharges of neuronal populations (“population spikes”) (Berger et al., 2005; Dimoka et al., 2008; Gholmieh et al., 2002) as well as dendritic shaft input to somatic membrane voltage output (Cook et al., 2007). How these results might relate to the information-rich spike patterns used for communication across brain circuits is uncertain. Important work was also done whereby system identification on spike patterns was conducted using *in vivo* recordings from CA3 (input stage) and CA1 (output stage), although during well-defined behaviors which restricts the set of input patterns (those associated with the behavior) and thus cannot produce the generalized kernels obtained with sufficiently exciting inputs in an *in vitro* model (Berger et al., 2012; Song et al., 2007, 2018). Moreover, correlations between the two subfields *in vivo* could in part reflect near simultaneous input to both regions from a third region such as the entorhinal cortex or septum, areas known to synchronize activity within the hippocampus. Nonetheless, the results provide special cases that must be satisfied by universal models. But assuming that reliable mathematical relationships for the EPSP-to-spike pattern transition can be developed, then it should be possible to assemble the diverse transfer functions for each of the links and nodes in the hippocampal circuit into a single predictive model. This would be a significant step toward the development of biologically realistic models of the brain.

## Data Availability

The raw data supporting the conclusions of this article will be made available by the authors, without undue reservation.
