# Supplementary material for: A concise mathematical description of signal transformations across the hippocampal apical CA3 to CA1 dendritic response
Source: Front Neural Circuits. 2026 Feb 12;19:1545031. doi: 10.3389/fncir.2025.1545031 (PMC12935924; doi:10.3389/fncir.2025.1545031)
Supplement: Supplementary file 1 [file Data_Sheet_1.pdf]

Methods

Animals

Experiments were conducted using 20 male mice (10 C57BL/6N) 2-3 months of age (**Supplementary Tables 1-2**); 10 animals were utilized for apical and 10 for basal recordings. Animals were group-housed (4-5 per cage), on a 12-hr light/dark cycle and with food and water *ad libitum*. All procedures were approved by the UC Irvine Institutional Animal Care and Use Committee and in line with the NIH guidelines for the Care and Use of Laboratory Animals.

Hippocampal slice electrophysiology

Animals were sacrificed between 10:00am-11:00am. Acute hippocampal slices were prepared as described previously [1]. Briefly, transverse slices of the middle third of the left hippocampus (360 µm thickness) were cut using a McIlwain chopper and collected in chilled artificial CSF (ACSF) which contained in mM (124 NaCl, 3 KCl, 1.25 KH_2PO_4, 1.5 $MgSO_4$, 26 $NaHCO_3$, 2.5 $CaCl_2$, and 10 dextrose). Slices were then transferred to the interface chamber kept at 31±1°C and had 60-70 mL/hr infusion of oxygenated ACSF. Slices were left for 1.5-2 hours to equilibrate prior to recording.

A stimulating electrode (twisted nichrome) was placed in the CA3-CA1 Schaffer collaterals and a glass recording electrode (2 M NaCl filled, 2-3 MΩ) was used to record extracellular field potentials from the CA1 dendrites. For apical dendritic recordings, the stimulating and recording electrodes were placed in the CA1b stratum radiatum with the stimulating electrode slightly offset towards the stratum pyramidale, and the recording electrode in the middle of the stratum radiatum [1]. In most animals, stimulation intensity (x_1) was set to induce a ~3.5-4 mV population-spike free fEPSP. For basal dendritic recordings, the stimulating and recording electrodes were placed in the CA1b stratum oriens with the recording electrode in the middle of

the stratum oriens and the stimulating electrode adjacent to it. In most animals, stimulation intensity (x_1) was set to induce a ~1 mV population-spike free fEPSP.

Recording protocol

For each animal, a 10-minute stable baseline session was recorded before experiment initiation. Baseline stimulation consisted of a single pulse every 20 seconds. After ensuring stable baseline responses, two or more 15-min sessions were recorded per animal. Slices were left to stabilize for 5 minutes without stimulation between the 15-min sessions. Stimulation patterns were random with spike times drawn from a Poisson distribution (lambda of 2 spikes/second) and amplitudes uniformly drawn from two values (x_1 and x_2: x_1 induced a 3-4 mV and ~1 mV fEPSP in the apicals and basals, respectively and x_2 was a fraction of x_1, **Table 1-2**). Recording duration and patterns were explored and determined through simulations such that they yielded accurate parameter estimates (**Supplementary Figure S2**).

Equipment

The stimulation signal *x(n)* was generated digitally in MATLAB 2018a (The MathWorks, Natick, MA), converted to an analog signal using 16-channel data acquisition hardware (DA-16, A-M Systems), which was then connected to a stimulus isolation unit (2200, A-M Systems) enabling the delivery of current to the stimulating electrode. DA-16 was also used to digitally record the delivered analog input *x(t)* and CA1 analog output *y(t)*, sampled at 10,000 Hz and saved in MATLAB. Both stimulus delivery and recording implemented through the DA-16 were interfaced using MATLAB and its Data Acquisition Toolbox for Measurement Computing.

Analysis: Simulations

Prior to initiation of data collection, simulations were conducted to aid in the appropriate experimental design for accurate estimates of 2nd order, 60-msec memory Volterra kernels.

Ground truth first- and second-order Volterra kernels were generated, using knowledge of the structure of these kernels (symmetric second-order kernel) and candidate CA1 dynamics (**Supplementary Figure 2A**). The ground truth first-order kernel was defined as an fEPSP. This is because for a linear system, *h(i)* is the response to a delta or spike input and the CA1 dendritic field generates an fEPSP to a spike input. Using these ground truth kernels, we tested the nature of the input and amount of data needed for accurate kernel estimation.

First, we found that increased randomness in the input increases the likelihood of tapping into the second-order structure. This is because such random input would likely include all possible τ, or ISIs, and kernel weights for all possible τ would be utilized and reflected in the output. Then, such kernel weights can be recovered with the input/output data. We therefore decided to deliver inputs at random times pulled from a Poisson distribution, which also mimicked the underlying distribution of neural spike timing (**Supplementary Figure 2B**). The Poisson parameter lambda was set to 2 spikes/second. A slow spike rate was chosen to prevent overstimulating the slice thereby affecting its dynamics during the recording and because this spike rate was used in prior work [2-4].

Second, we found that a binary input leads to estimation bias due to multicollinearity – where the first-order kernel and the diagonal elements of the second-order kernel become coupled. This is because the first-order kernel provides weights for individual input values and the diagonal of the second-order kernel provides weights for the square of such values. With a binary input (0 and a non-zero value), the square of the input vector becomes a linear combination of it leading to multicollinearity and estimation bias. We therefore randomly and uniformly drew the spike amplitude from two values, x_1 and x_2 (x_1 induced ~3-4 mV and ~1mV responses in the apicals and basals, respectively, and x_2 was a fraction of x_1, see **Tables 1-2**).

Finally, for the desired model order – a 60 msec kernel length and up to a second-order kernel, we quantified the normalized estimation error as a function of data duration (**Supplementary Figure 2C-D**). The error reached a plateau of 0 with 13-15 minutes for a single session.

Analysis: Data curation and preprocessing

Data curation and preprocessing were done offline in MATLAB. The recorded input signal was used to identify the input timestamps. The detected timestamps were then used to remove stimulation artifacts from the CA1 record. Local points (17 data points ~1.7 ms; 3 time points (tp) before and 14 sample points after) around the artifact peak were replaced by propagating the CA1 signal immediately before the artifact using the slope of the signal at the boundaries of the artifact ($\frac{y_{14\,tp} - y_{3\,tp}}{17}$) plus noise pulled from a normal distribution with 0 mean and the standard deviation of the CA1 artifact free record weighted by 1/16. Then, other large amplitude measurement noise data points were replaced by the mean of the stimulation artifact-free recording plus noise pulled from a normal distribution (0 mean and the standard deviation of the CA1 artifact free record). The data was then downsampled to 1000 Hz. All individual animal kernel estimates took place subsequently using this data (non-normalized).

For group analysis, additional preprocessing was implemented and consisted of within animal 1) pre-stimulation normalization and 2) normalization of fEPSP amplitude relative to the mean fEPSP induced by x_1. Pre-stimulation normalization accounts for slow drifts in the data, which offset the starting value of the induced fEPSP. Each value following a stimulus was z-score normalized relative to the mean and standard deviation of a 100 msec (1000 data points, before downsampling) period prior to stimulation that does not overlap with a preceding CA1 response (after an 80 msec duration of a prior response) from a prior stimulus. For closely occurring successive stimulations, however, this baseline period did not exist. Therefore, successive

stimuli occurring proximally in time shared the same pre-stimulus baseline period which occurred before the train.

After pre-stimulus baseline normalization, all values were normalized to the negative peak of the mean fEPSP trace induced by x_1. This enabled all $y(n)$ output values to be bound between 0 and ~-1 for all animals. Additionally, this enabled normalization of the input signal across animals; whereby the x_1 inducing ~ -1 normalized response was arbitrarily mapped to 1 for all animals. Then, x_2 became simply equal to the original fraction used during the experiment – either 0.5, 0.75, or 0.8.

Analysis: Kernel estimation

For each 15-min session recorded per animal, the last 12-min (most stationary subset) was utilized to obtain individual animal estimates for subsequent group analysis. This choice accounted for the tradeoff between stationarity and sufficient data for accurate estimation (**Supplementary Figures 2**). The session exclusion criteria included: 1) measurement noise preventing the stimulation detection, 2) non-invertibility of the design matrix, 3) recording error, 4) lack of slice stability (non-stabilizing fEPSP slope/peak values), and 5) high amplitude, temporally sustained measurement noise.

The curated CA1 output signal was used as the $y(n)$ vector, and an equal length spike vector, $x(n)$, was re-arranged into a matrix, X_{design} such that the kernel estimation is reformulated into a least squares regression problem $y(n) = X_{\text{design}} * h_{all}(n)$ and solved using the pinv function in MATLAB. $h_{all}(n)$ contains all kernels (both first- and second-order) in vector format. It is important to note that in generating the design matrix, we took advantage of the symmetry of the second-order kernel and only estimated the diagonal and upper triangular elements of the kernel to reduce the number of unknown parameters.

Analysis: normalized prediction error

Normalized prediction error was quantified as the sum of the squared error (predicted - true) normalized by the sum of the squared true output [5]. This measure was calculated for stimulation epochs (each stimulation time point and the following 60 msec data). Such epochs are ones where non-zero predictions were made, otherwise the system is assumed to be at rest (predictions for system output equal 0).

$$normalized\ prediction\ error = \sum_{stim\ epoch\ (t)\ timepoints} \frac{(y_{(t)\ predict} - y_{(t)\ true})^2}{{y_{(t)\ true}}^2}$$

Analysis: h(k,m) pruning

For each dendritic domain, a pruned version of *h(k,m)* was obtained. Pruning was done to remove coefficients that were near 0 and thereby obtain a sparser model that performs similarly to the full model. The pruning was done by examining the distribution of values for each entry in *h(k,m)* across sessions. Only entries with consistently large positive weights (large positive mean divided by standard deviation) and consistently large negative weights (large negative mean divided by the standard deviation) were kept, and the others were removed. This was done by gradually increasing the percentile thresholds for mean/standard deviation upper and lower bound cutoffs. For each threshold, pruned model performance (prediction error) was tracked. The final pruned models (retained apical entries: lower than the 60th and higher than the 80th percentiles; retained basal entries: lower than the 65th and higher than the 87th percentiles) were chosen such that a high degree of pruning is achieved with no significant difference in prediction accuracy compared to the full model. For both dendritic domains, *h(k,m)* entry value distribution was skewed to the left (higher probability of negative weights). For this reason, the lower bound is as large as the 60-65th percentile; values below these percentiles were negative, values between 60-65th and 80-87th were primarily 0, and values above the 80-87th percentiles were positive for the apicals and basals, respectively.

Analysis: paired pulse predictions

Paired pulse predictions were examined in each node to test whether the system is indeed second order (**Supplementary Figure 4-7** for basals and **Supplementary Figure 8-11** for basals). This was done by detecting from all recorded sessions all paired pulses with ISIs anywhere from 1 to 60 msec, irrespective of other pulses occurring in-between.

Analysis: differences in kernel estimates between nodes

Significant testing for differences in *h(k,m)* kernel values between the apical and basal dendritic domains were identified using cluster-based permutation testing [6]. Briefly, this involved calculating a t-statistic in each entry, between the apical and basal *h(k,m)* 2-D observed matrices (n = 20 for the apicals and n = 37 for the basals), thereby generating an *observed t-map*. The observed t-map was then compared to a null distribution of t-maps generated over 1000 unpaired permutations. In each permutation, condition labels were shuffled such that all *h(k,m)* matrices estimated from apical and basal recordings were randomly allocated to two fake groups with the same discrepancy in sample size (n = 20 for condition 1 and n = 37 for condition 2, hence unpaired permutations). For each entry, the observed t-value was compared to the null t-value in the same entry, in terms of the number of standard deviations the observed value was greater or less than the mean of the null t-value, thereby generating a z-map. The z-map was then converted to a *p*-map in which a *p*-value for each entry was obtained. To correct for multiple comparisons (number of entries tested), clusters of contiguous entries with $p < 0.05$ were identified and compared to the null-distribution cluster size. Observed clusters with sizes larger than the 95th percentile of those from the null distribution were considered significant after correction for multiple comparisons.

Significant testing for differences in *h(i)* between the two regions was conducted using the same procedure but applied on a 1-D vector of entries rather than a 2-D matrix of entries [6].

Analysis: Statistical analyses

Significance testing on prediction error was done using non-parametric paired permutation testing with 100 permutations. Significance testing on differences in true-versus-predicted paired pulse responses was done using the 1-D cluster-based permutation testing discussed in the prior section (‘*Analysis: differences in kernel estimates between nodes*’).

References

1. Wang, W., et al., *Memory-Related Synaptic Plasticity Is Sexually Dimorphic in Rodent Hippocampus.* J Neurosci, 2018. **38**(37): p. 7935-7951.
2. Sclabassi, R.J., et al., *Nonlinear systems analysis of the hippocampal perforant path-dentate projection. I. Theoretical and interpretational considerations.* J Neurophysiol, 1988. **60**(3): p. 1066-76.
3. Berger, T.W., et al., *Nonlinear systems analysis of the hippocampal perforant path-dentate projection. II. Effects of random impulse train stimulation.* J Neurophysiol, 1988. **60**(3): p. 1077-94.
4. Berger, T.W., et al., *Nonlinear systems analysis of the hippocampal perforant path-dentate projection. III. Comparison of random train and paired impulse stimulation.* J Neurophysiol, 1988. **60**(3): p. 1095-109.
5. Dimoka, A., et al., *Modeling the nonlinear properties of the in vitro hippocampal perforant path-dentate system using multielectrode array technology.* IEEE Trans Biomed Eng, 2008. **55**(2 Pt 1): p. 693-702.
6. Cohen, M.X., *Analyzing neural time series data : theory and practice.* 2014.

Supplementary Material

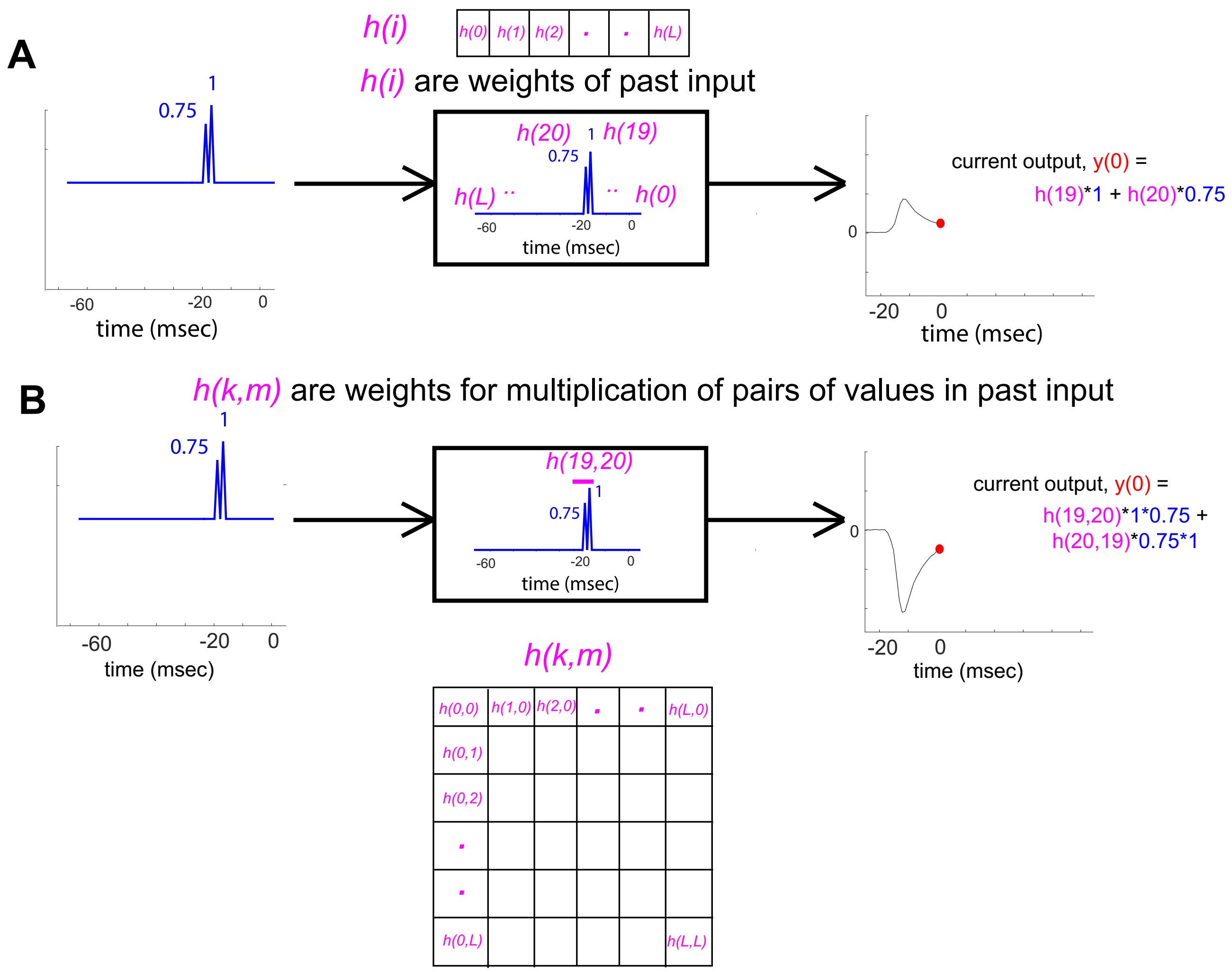

Supplementary Figure 1. Nonlinear system identification

A-B, Schematic representation of how *h(i)* (A) and *h(k,m)* (B) reflect system linear and second-order nonlinear operations, respectively. Blue traces preceding the arrow are inputs, black traces following the arrow are outputs and the blocks represent the system. System operations are reflected by the kernels *h(i)* and *h(k,m)* which are applied on incoming inputs to yield instantaneous output values. **A,** The term *h(i)*, the first-order kernel, is a vector of weights applied on current and past input values (up to L time-points in the input record) that contribute to the current output value (red circle). **B,** The term *h(k,m)*, the second-order kernel, is represented by a symmetric matrix; its τ's diagonal slices reflect how the output is influenced by

products of neighboring input values τ timepoints apart occurring up to L samples in the past.

Note: Output traces in A and B are not exact, but approximations to serve as an illustration.

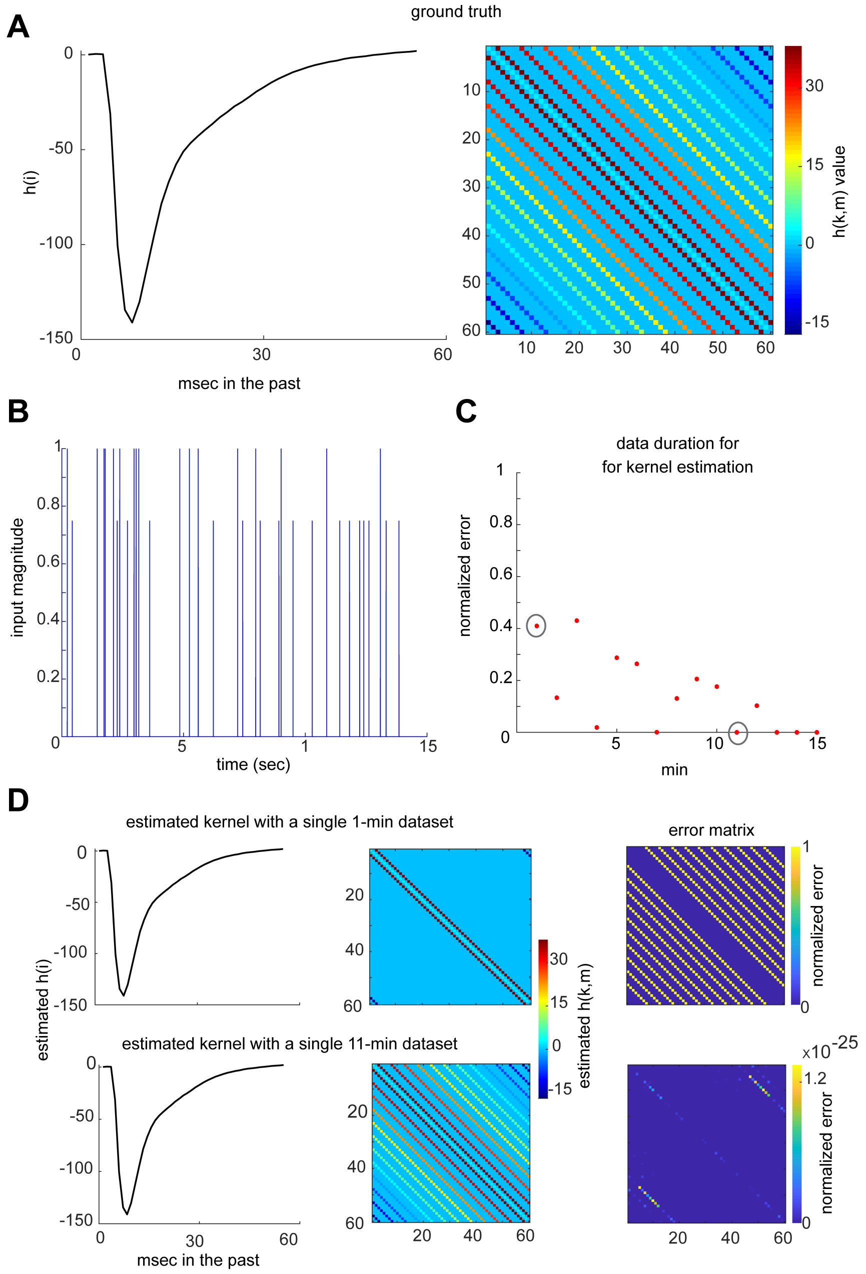

Supplementary Figure 2. Simulations to identify recording duration and input type needed for accurate and unbiased kernel estimation.

A, Artificially generated ground truth first (left) and second (right) order kernels. **B,** example of nonbinary (x_1 =1 or x_2 = 0.75* X_1) input with ISI from a Poisson distribution and spike amplitude from a uniform distribution. **C,** Normalized estimation error as a function of data duration. **D,**

Example kernel estimates (left) and *h(k,m)* error matrix (right) using 1 minute (top) and 11 min (bottom) data, whose total errors are circled in **C.**

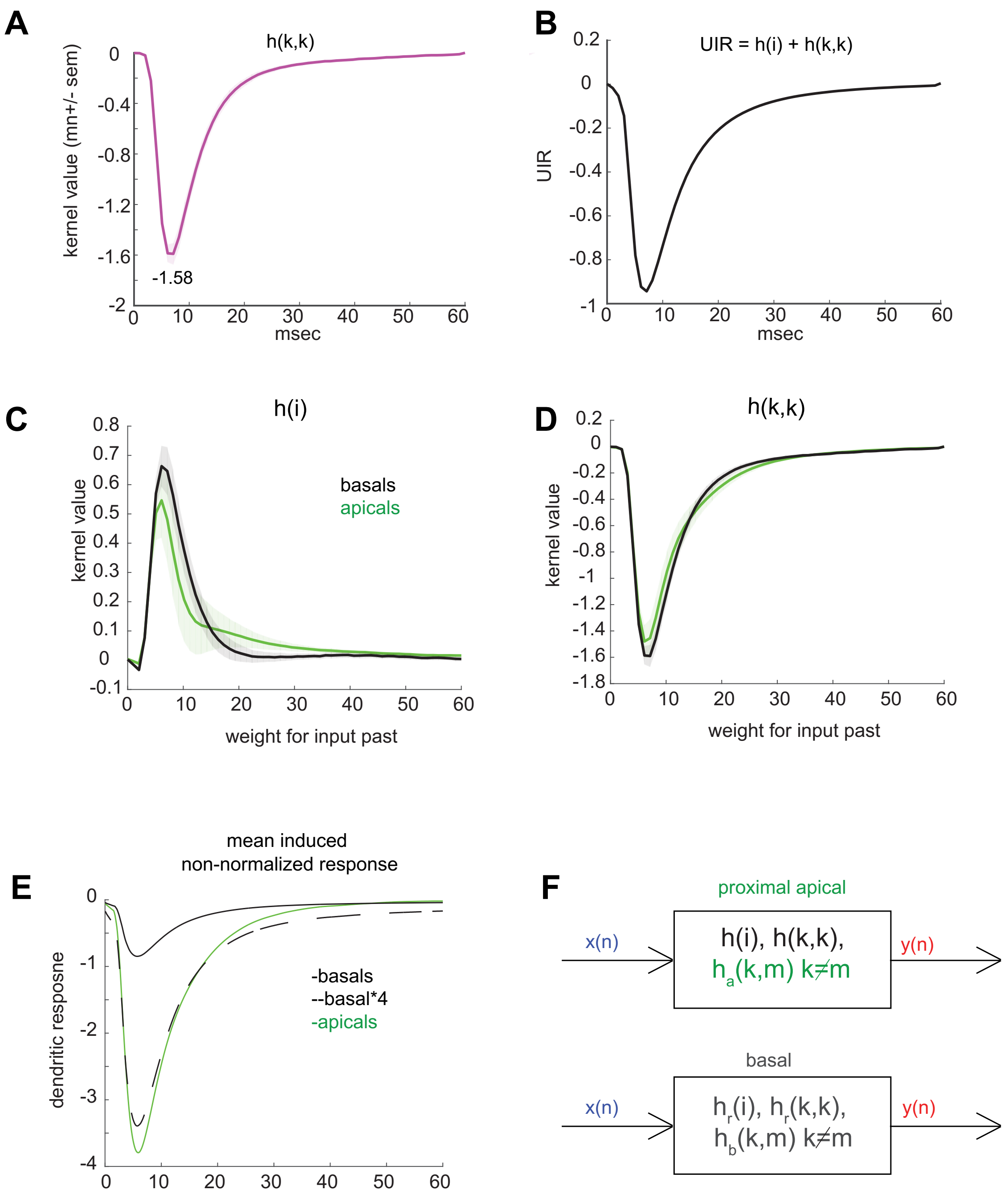

Supplementary Figure 3. Aspects of the basal dendritic system transfer function and its relation to the apical system.

A, Session mean *h(k,m)* main diagonal slice, *h(k,k)*. **B,** Session mean system unit impulse response (UIR). **C-D,** Session mean **(C)** *h(i)* and **(D)** *h(k,k)* estimates for the apical and basal systems (n = 20 and n = 37 sessions pooled from 10 animals per region, respectively). No significant *h(i)* nor *h(k,k)* differences were observed between the two dendritic domains (cluster-based permutation testing (CBPT), $p > 0.05$). **E,** Group mean induced response y_1 to all high amplitude input pulses x_1 aligned to the time of the input pulse and averaged for apical (green) and basal (black) recordings (n = 14,049 trials and n = 26,308 trials pooled from all sessions per region, respectively). Note the differences in the scaling of the raw induced fEPSP (before magnitude normalization). Dashed black line is a basal waveform rescaled by a factor of 4. **F,** Block diagram representation of transfer functions for the proximal apical (top) and basal (bottom) systems. $h_r(i)$ and $h_r(k,k)$ are rescaled versions of the same kernel waveforms for the apicals *h(i)* and *h(k,k)*, yielding a compressed basal fEPSP. $h_a(k,m)$ and $h_b(k,m)$ are the off-diagonal weights of the apical and basal *h(k,m)* nonlinear operations, respectively, which are known to differ (**Fig. 8A**). Error shades represent standard error of the mean across sessions.

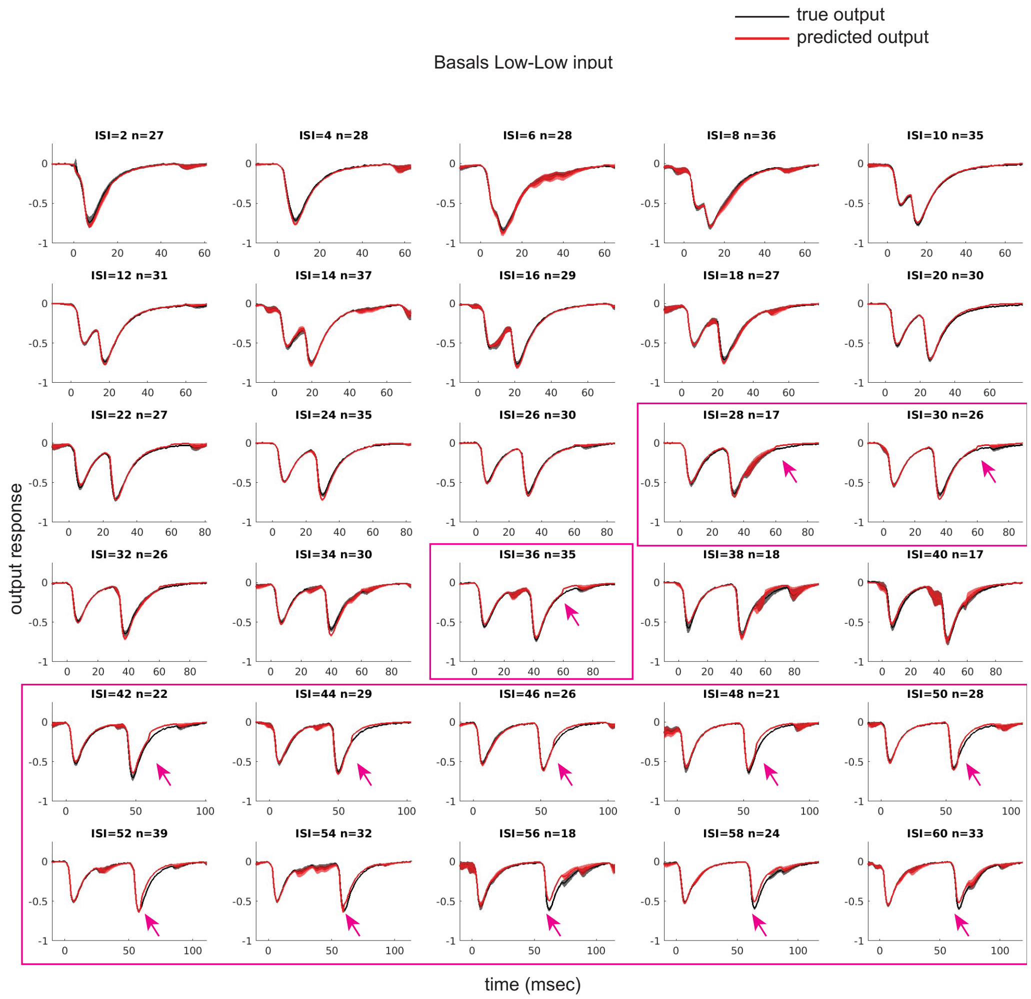

Supplementary Figure 4. Paired pulse predictions for the basal dendrites (low magnitude for both pulses)

True (black) and predicted (red) basal dendritic responses for paired pulse inputs with ISIs ranging from 2-60 msec in steps of 2 msec. For each plot, the ISI and number of trials used to generate the mean traces are indicated. Cluster-based permutation testing (CBPT) was performed to identify time segments that significantly differed between the true and predicted conditions (1000 permutations). CBPT was only performed on the 60-msec time period after the first pulse since predicted time points after such periods are the model's response to the second pulse treating it as an isolated pulse (model has no memory for first pulse). ISIs for which

significant differences were found are indicated by black boxed plots and the time duration that significantly differed between the true and predicted traces is indicated with a green line. No significant differences were found between the true and predicted responses for all ISIs plotted. Magenta boxed plots are a demonstration that system memory is larger than 60 msec. In those examples, the model's predicted traces deviate from the CA1's true response starting at the 60th msec onward, since at this point, the predicted output response is generated by operations on the second pulse alone while the CA1's true response accounts for both pulses (retained memory for the first pulse that occurred over 59 msec in the past). SEM reflects standard error of the mean across pooled trials with a given ISI from all sessions.

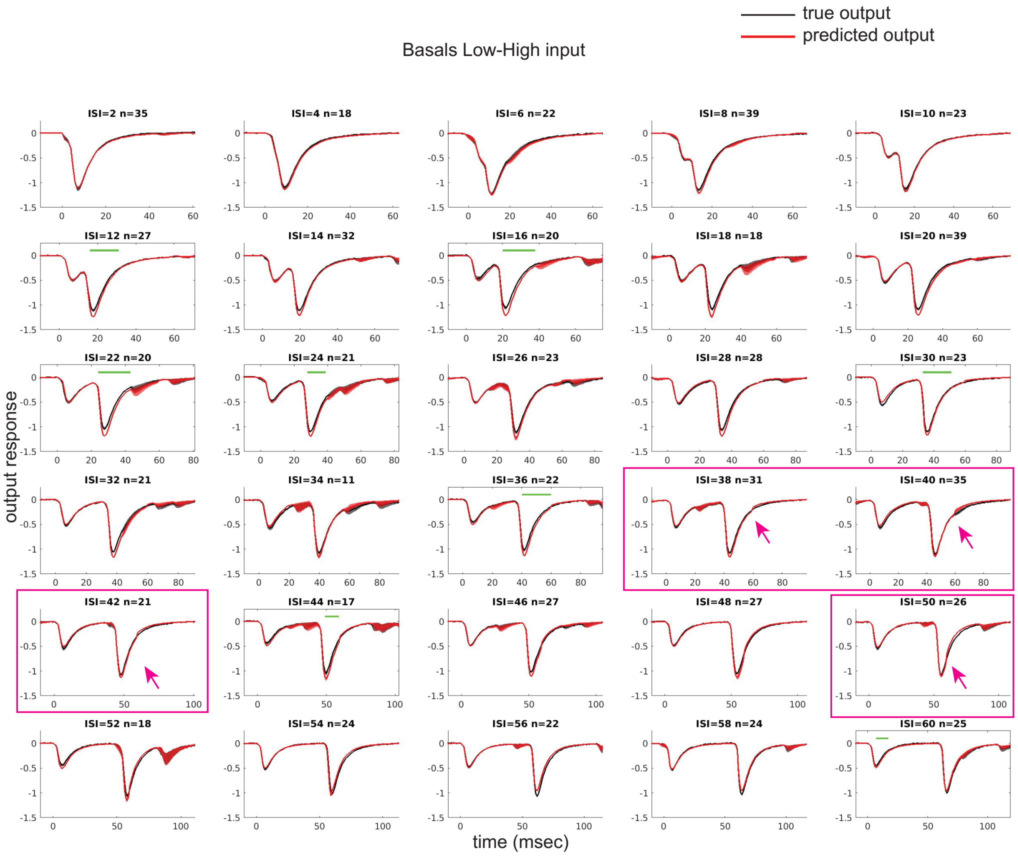

Supplementary Figure 5. Paired pulse predictions for the basal dendrites (low magnitude followed by high magnitude paired pulses)

Same as Supplementary Figure 4, but for low followed by high magnitude paired pulses. ISIs for which significant differences were found are indicated by black boxed plots (black square) and the time duration that significantly differed between the true and predicted traces is indicated with a green line.

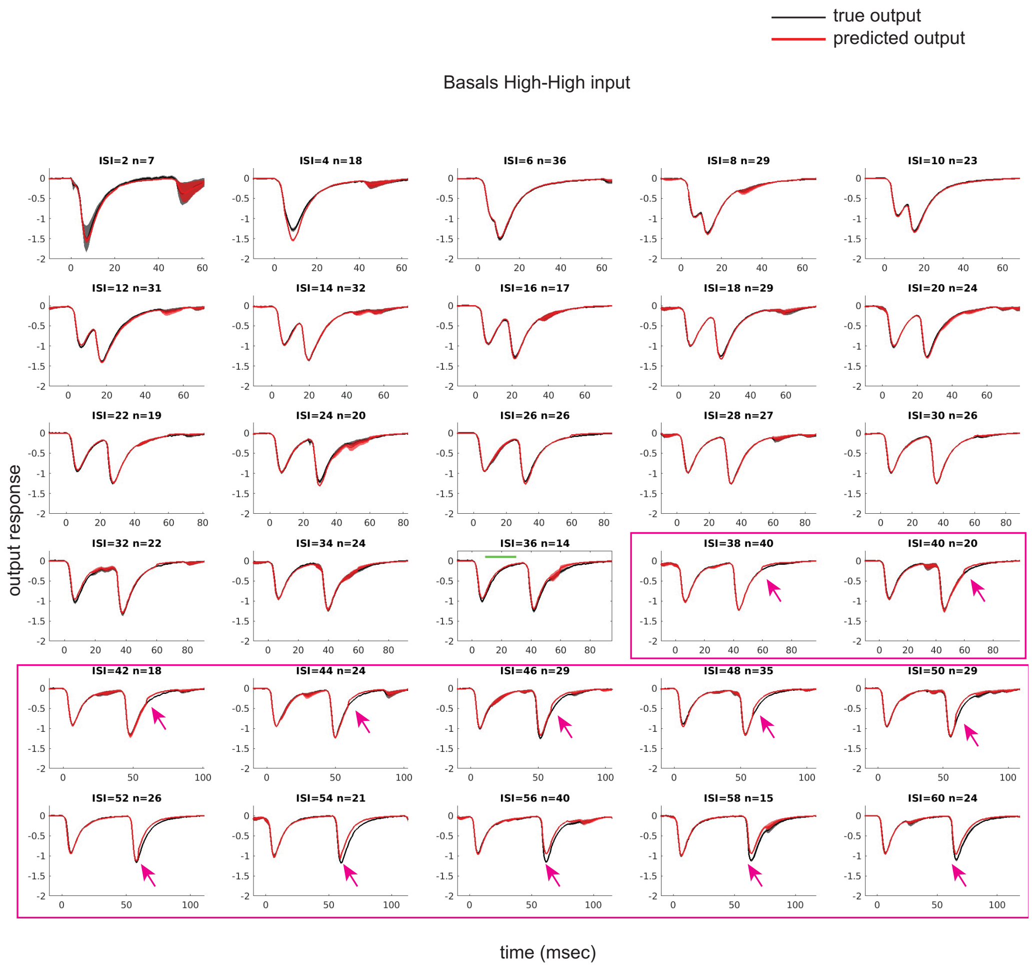

Supplementary Figure 6. Paired pulse predictions for the basal dendrites (high magnitude for both pulses)

Same as Supplementary Figure 4, but for high magnitude pulses.

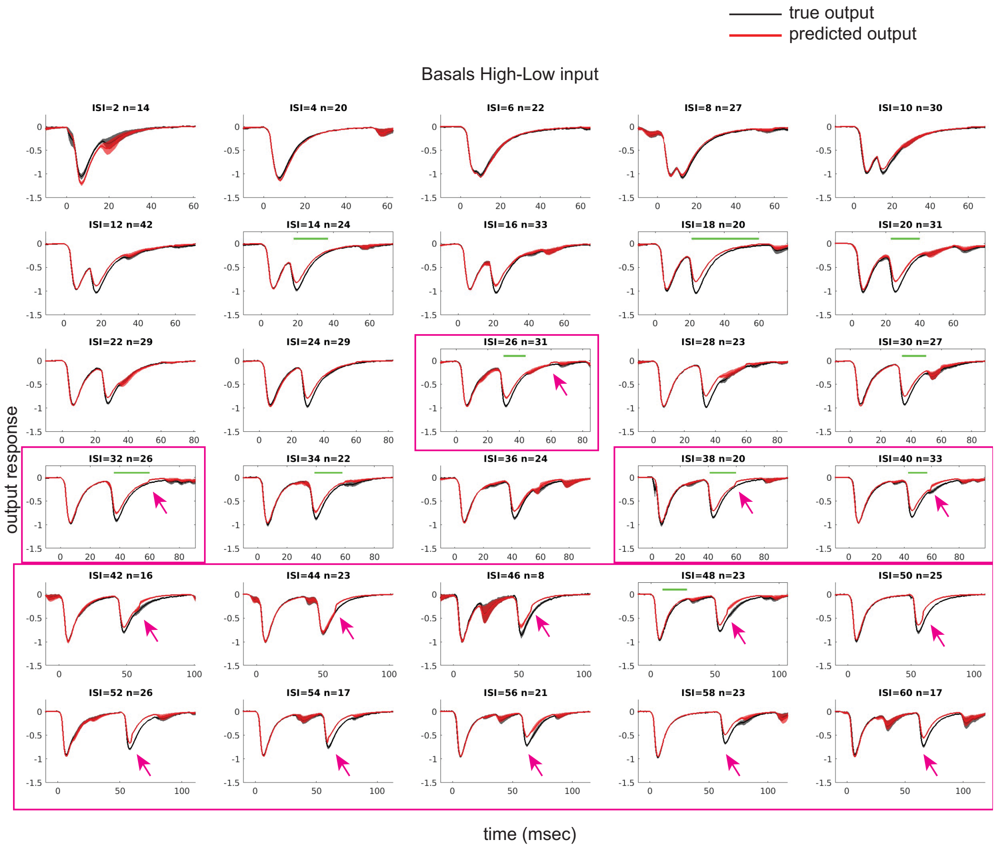

Supplementary Figure 7. Paired pulse predictions for the basal dendrites (high magnitude followed by low magnitude paired pulses)

Same as Supplementary Figure 4, but for high followed by low magnitude paired pulses.

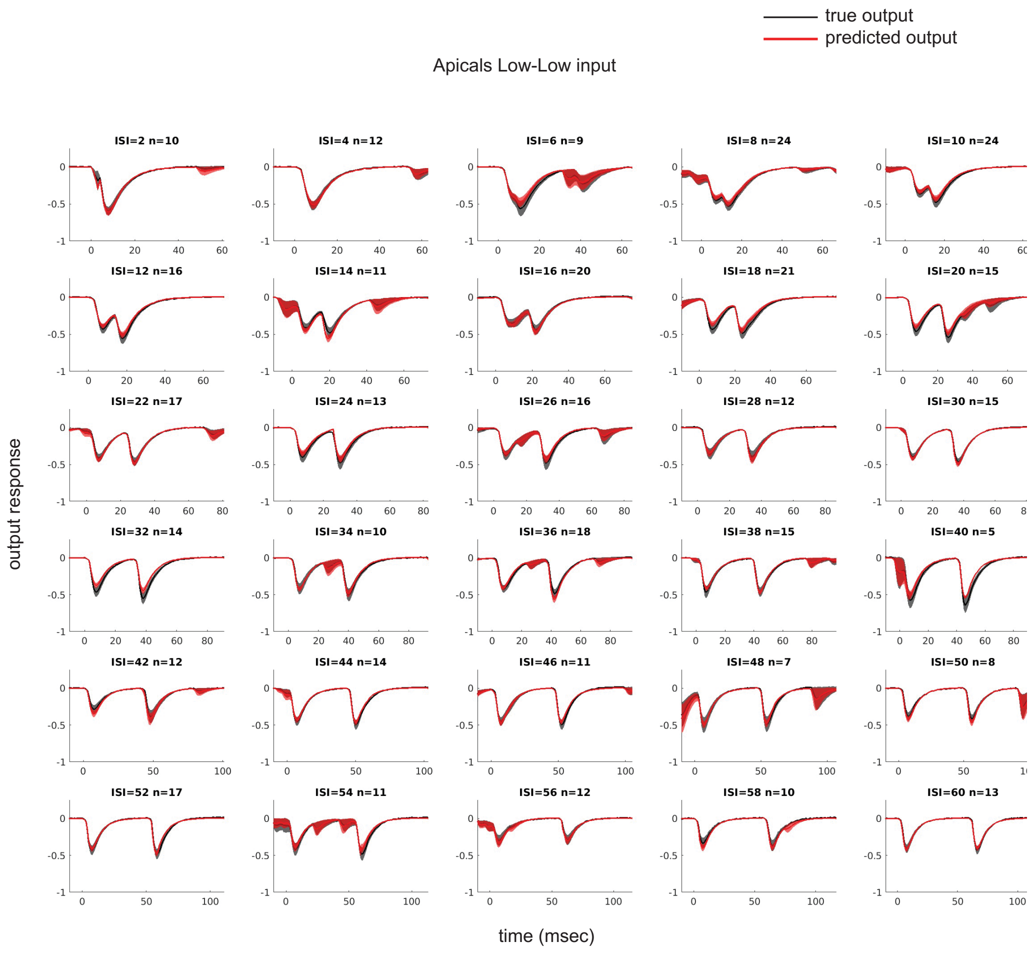

Supplementary Figure 8. Paired pulse predictions for the apical dendrites (low magnitude for both pulses)

Same as Supplementary Figure 4, but for the apical dendrites.

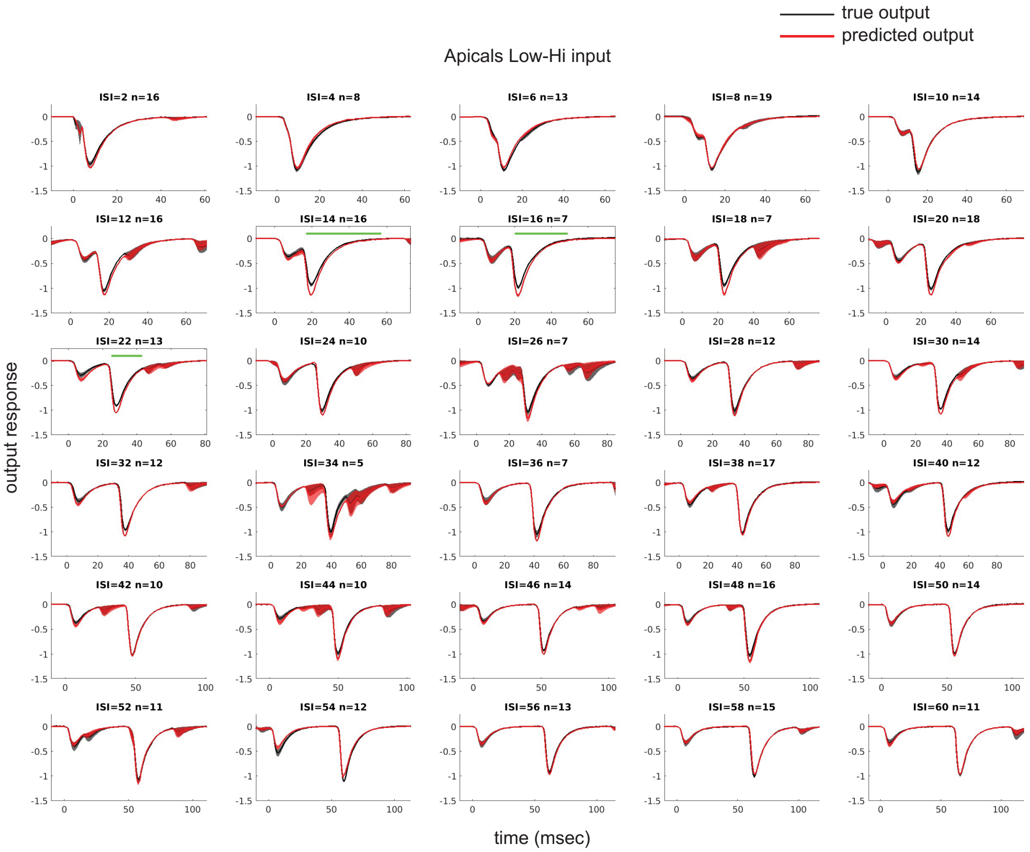

Supplementary Figure 9. Paired pulse predictions for the apical dendrites (low magnitude followed by high magnitude paired pulses)

Same as Supplementary Figure 5, but for the apical dendrites.

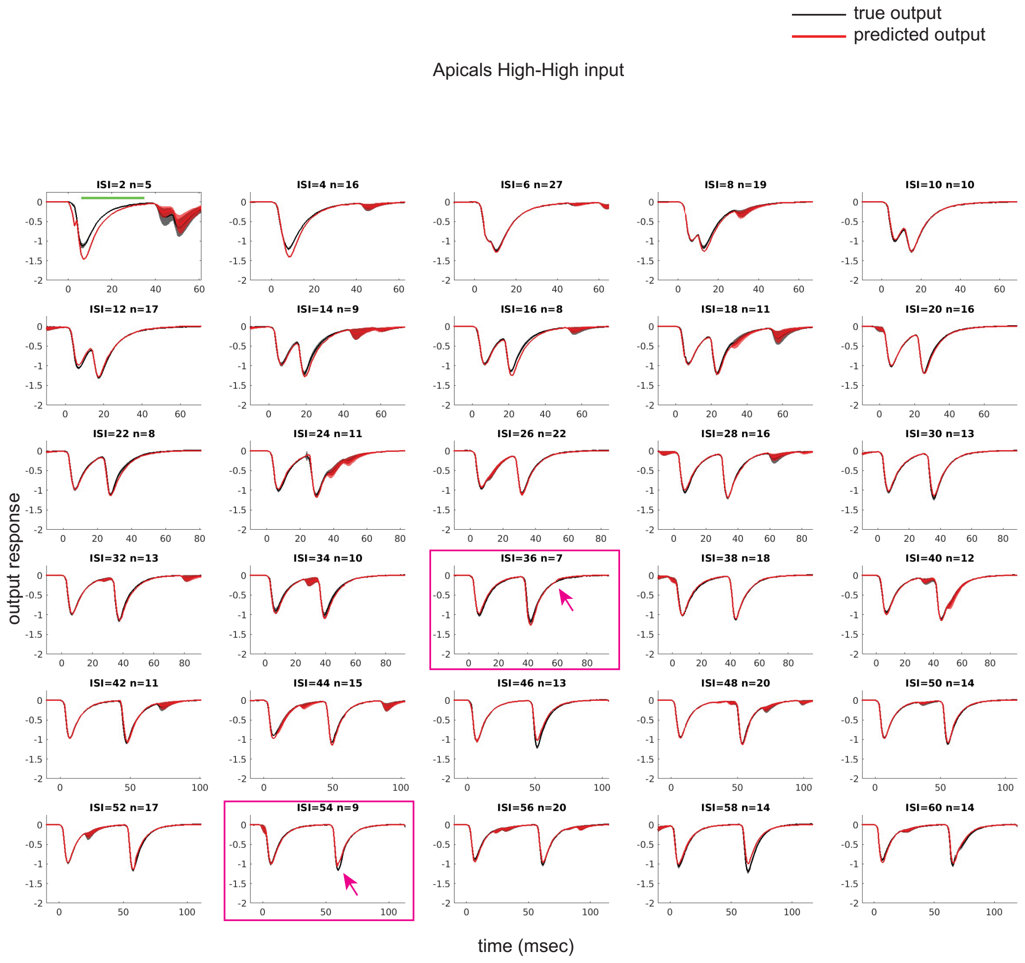

Supplementary Figure 10. Paired pulse predictions for the apical dendrites (high magnitude for both pulses)

Same as Supplementary Figure 6, but for the apical dendrites.

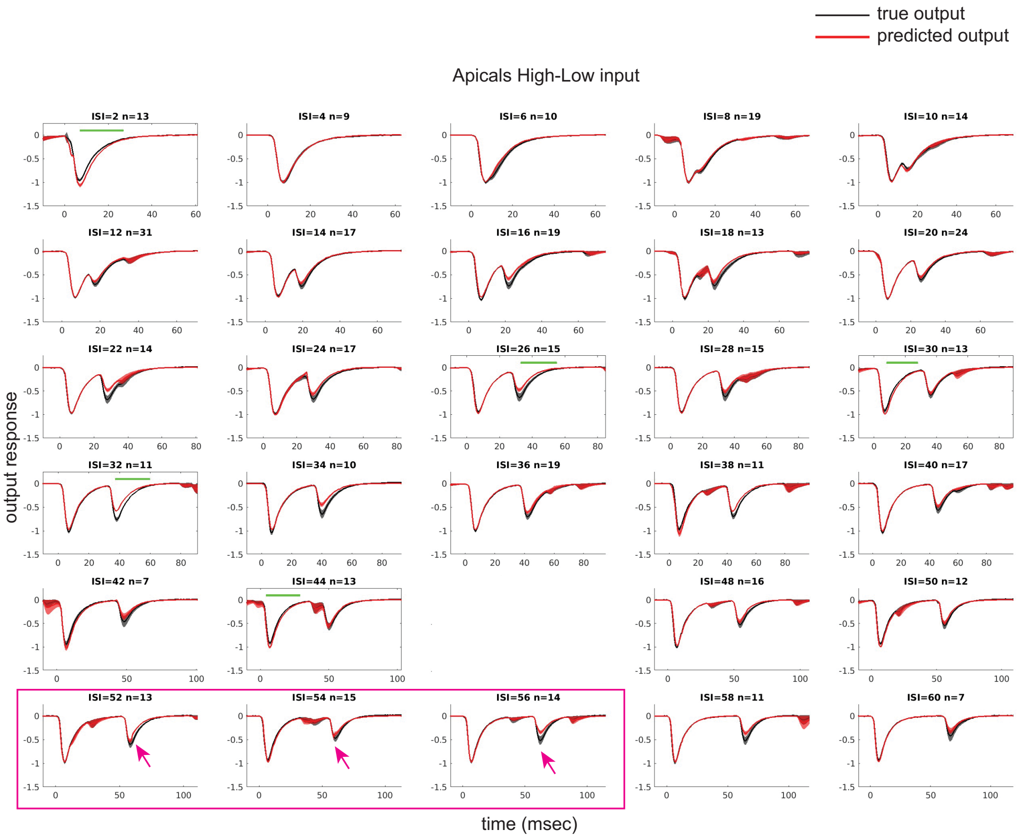

Supplementary Figure 11. Paired pulse predictions for the apical dendrites (high magnitude followed by low magnitude paired pulses)

Same as Supplementary Figure 7, but for the apical dendrites.

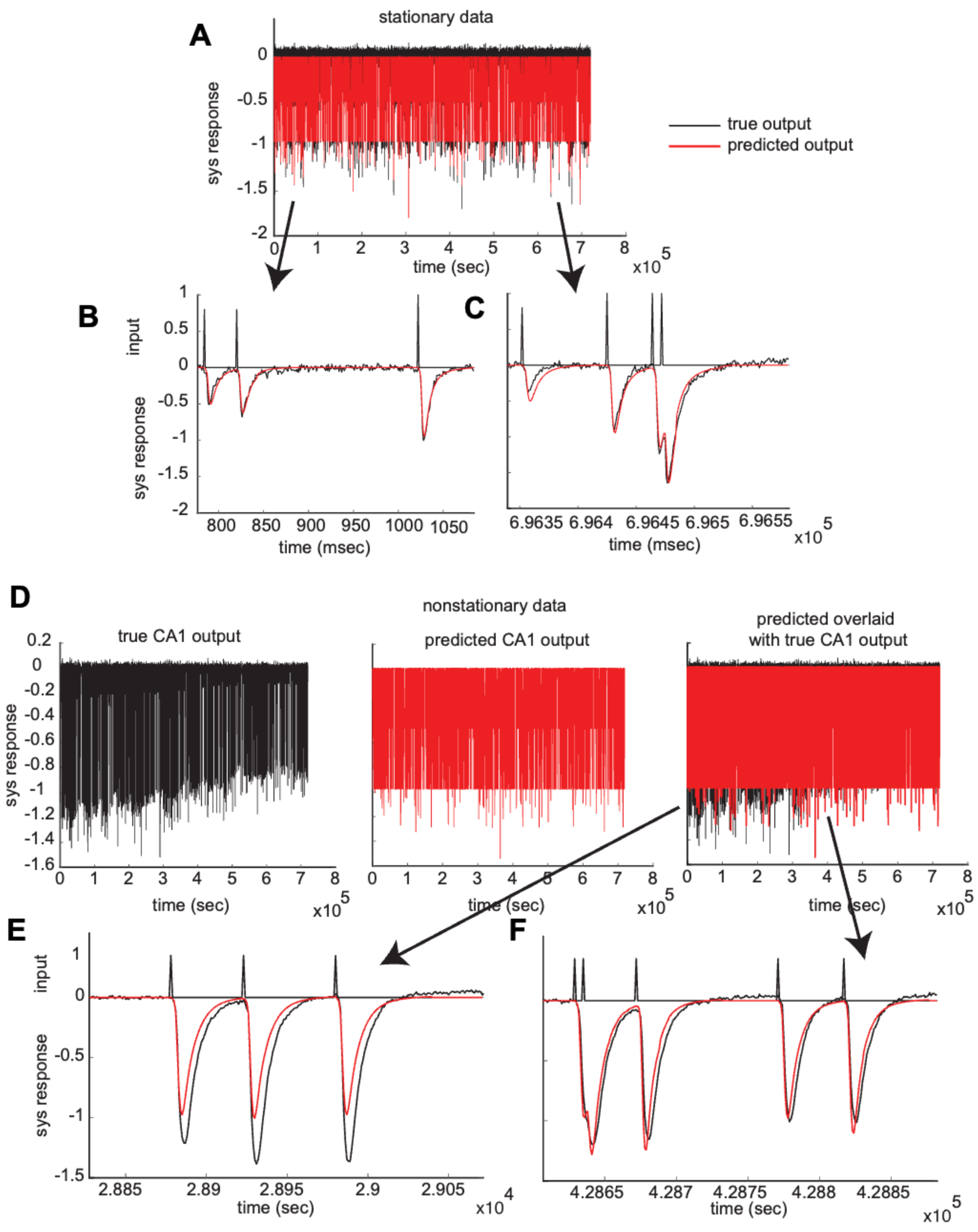

Supplementary Figure 12. Nonstationarity is a source of prediction error

A, Example of a stationary (constant response magnitude as a function of time) recording from the basal dendrites. Black and red traces reflect true and basal-transfer-function predicted

responses, respectively. **B-C,** True output is within the range of the predicted output for segments extracted from both the beginning (B) and end (C) of the recording. **D,** Example of a nonstationary (decreasing response magnitude as a function of time) recording from the apical dendrites (left panel), predicted output (stationary by definition, middle panel), and both predicted and true outputs overlaid (right panel). Note the true outputs are outside (more negative) the prediction range, in the first half of the recording (right panel). **E-F,** True output is outside (E) and within (F) the range of the predicted output for segments extracted from the beginning (E) and middle (F) of the recordings, respectively. It is important to note that data was not excluded for nonstationary, for both model estimation and testing (see exclusion criteria list under 'Analysis: kernel estimation' section in the methods). The influence of nonstationarity was minimized analytically by indexing only the last 12 minutes of all 15-minute recording sessions for all data analyses.

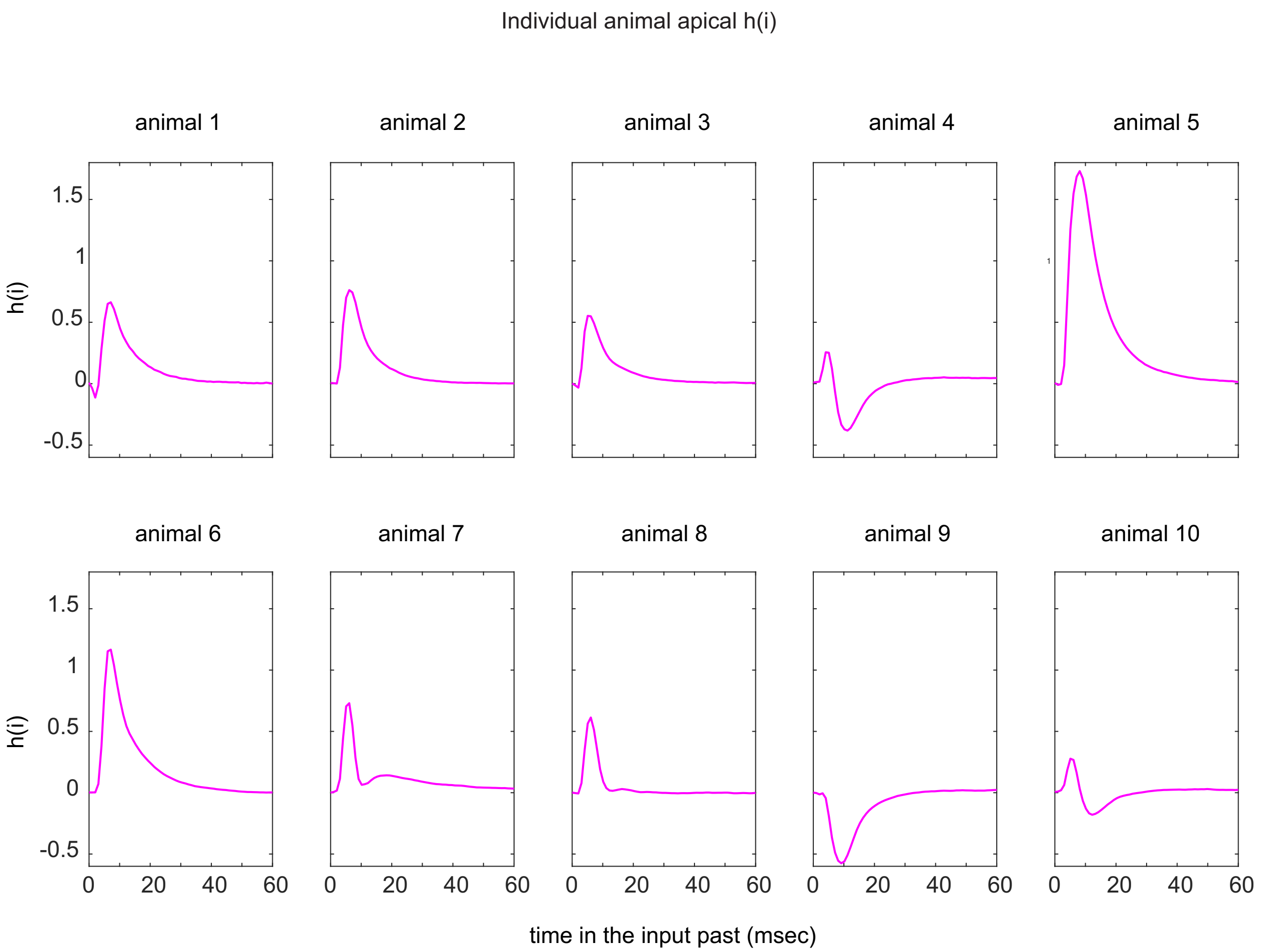

Supplementary Figure 13. Individual animal *h(i)* estimates

Each trace is an average across session (last 12-minutes) estimates recorded in the same animal (see Table 1).

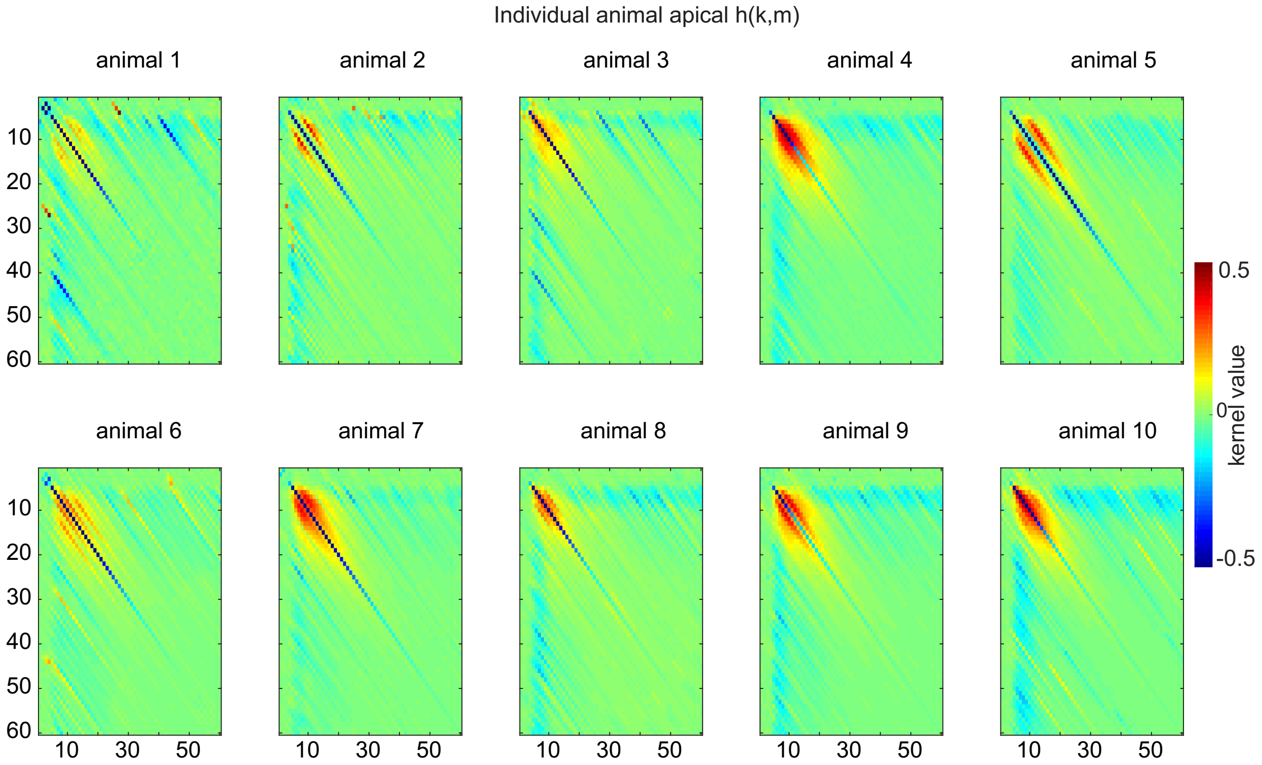

Supplementary Figure 14. Individual animal *h(k,m)* estimates

Each matrix is an average across session (last 12-minutes) estimates recorded in the same animal (see Table 1).

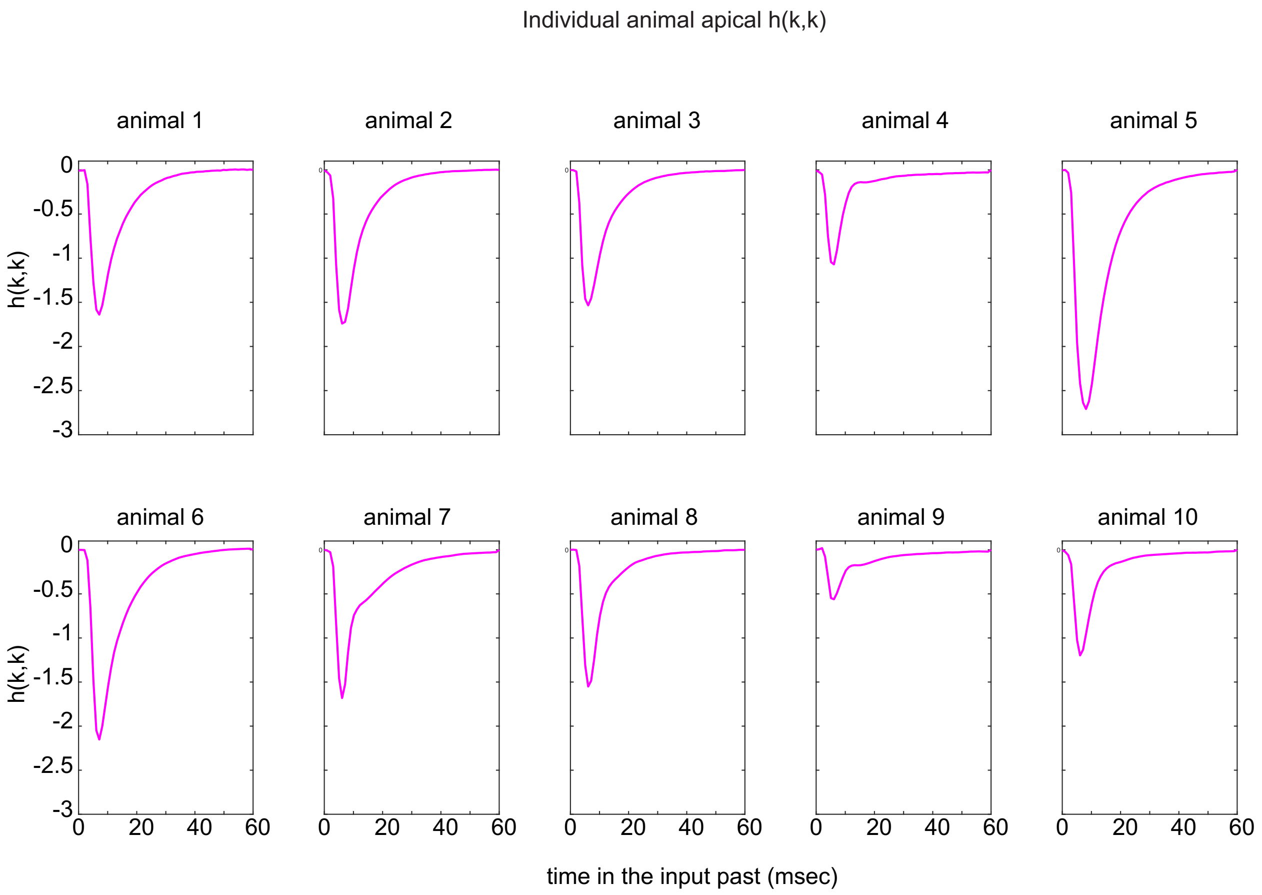

Supplementary Figure 15. Individual animal *h(k,k)* estimates

Each trace is an average across session (last 12-minutes) estimates recorded in the same animal (see Table 1).

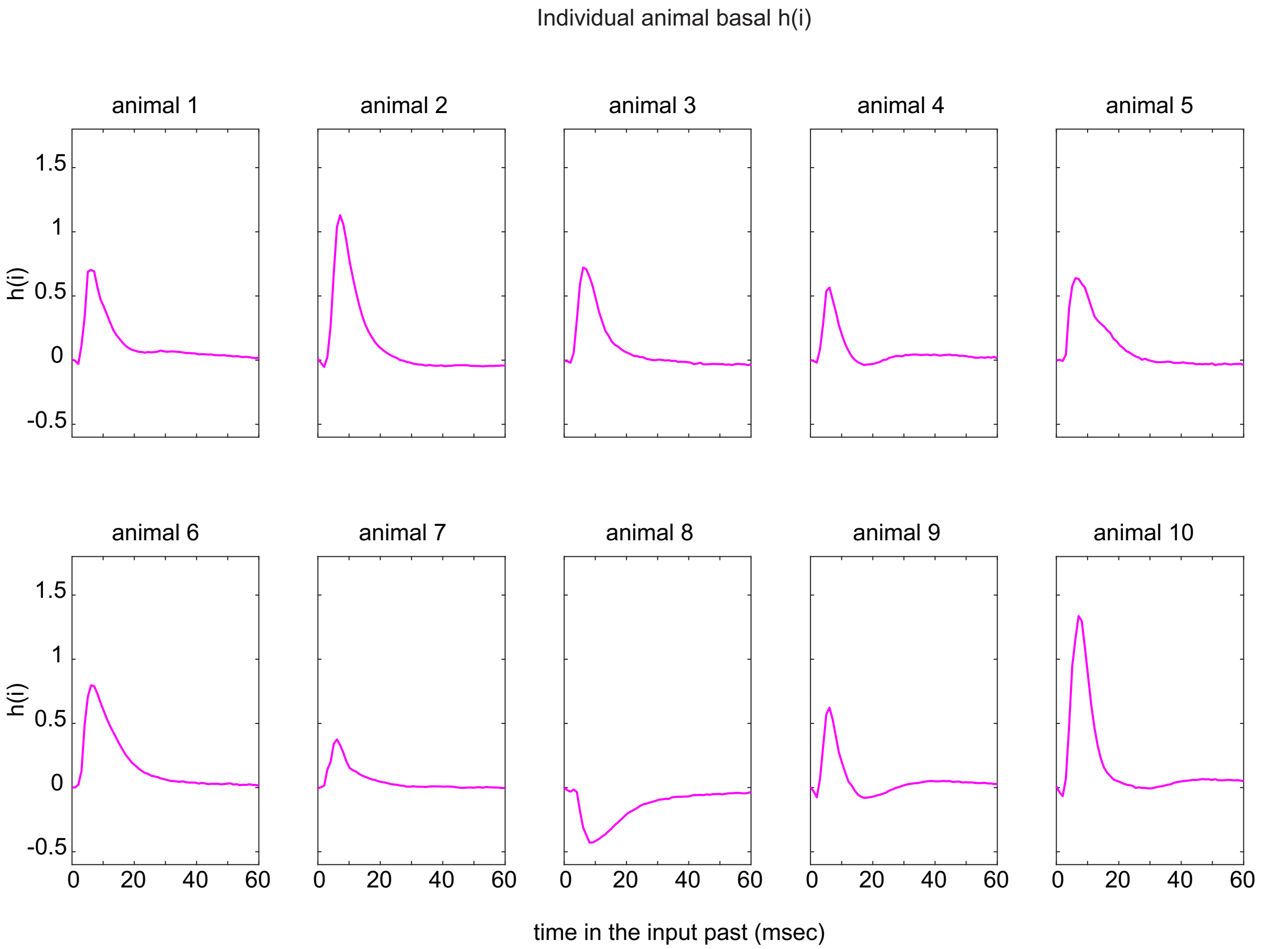

Supplementary Figure 16. Individual animal *h(i)* estimates

Each trace is an average across session (last 12-minutes) estimates recorded in the same animal (see Table 2).

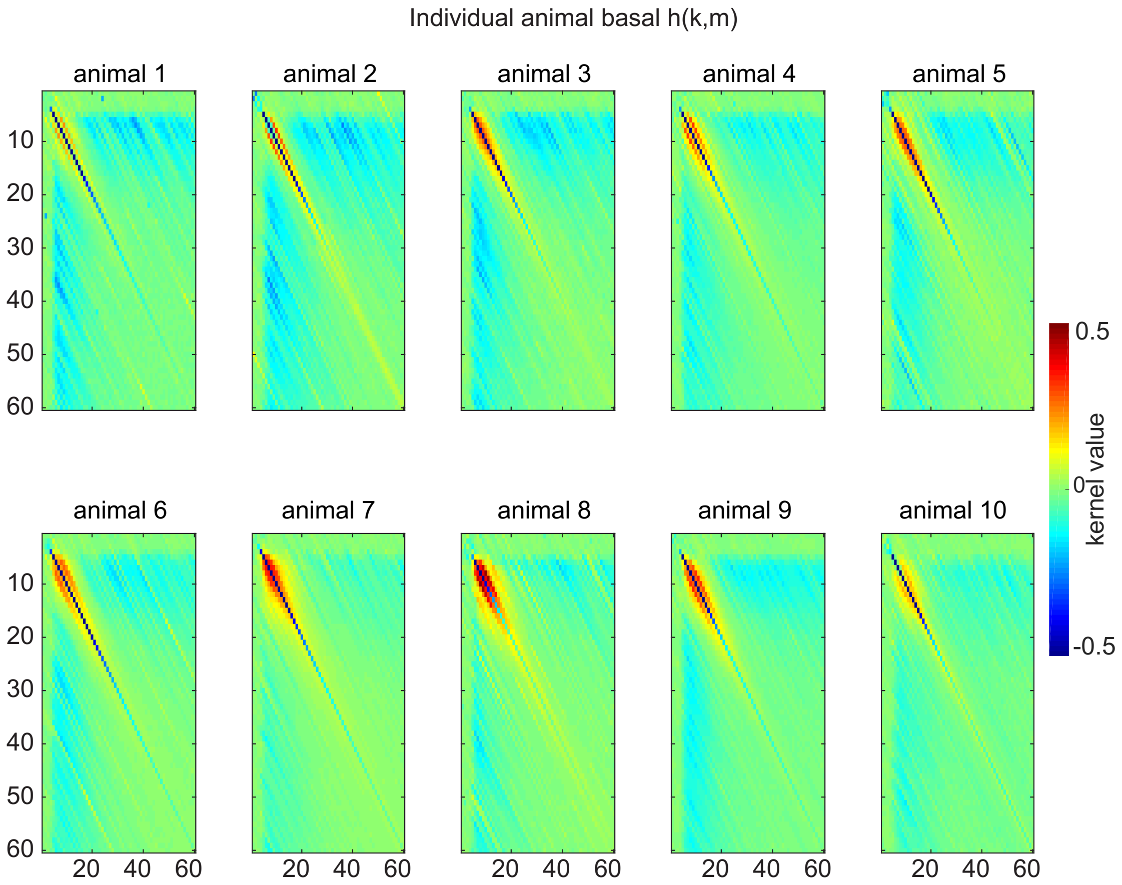

Supplementary Figure 17. Individual animal *h(k,m)* estimates

Each matrix is an average across session (last 12-minutes) estimates recorded in the same animal (see Table 2).

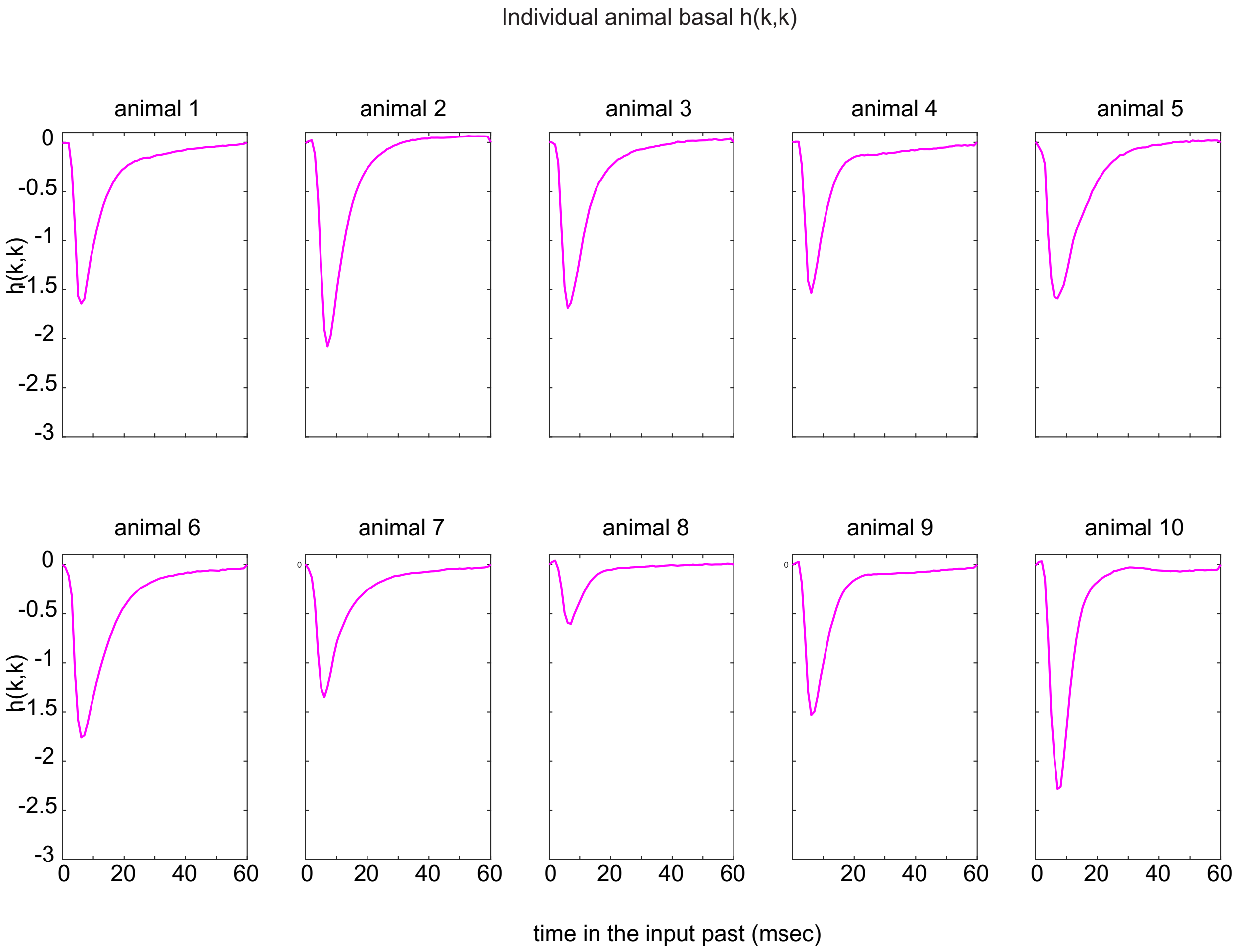

Supplementary Figure 18. Individual animal *h(k,k)* estimates

Each trace is an average across session (last 12-minutes) estimates recorded in the same animal (see Table 2).

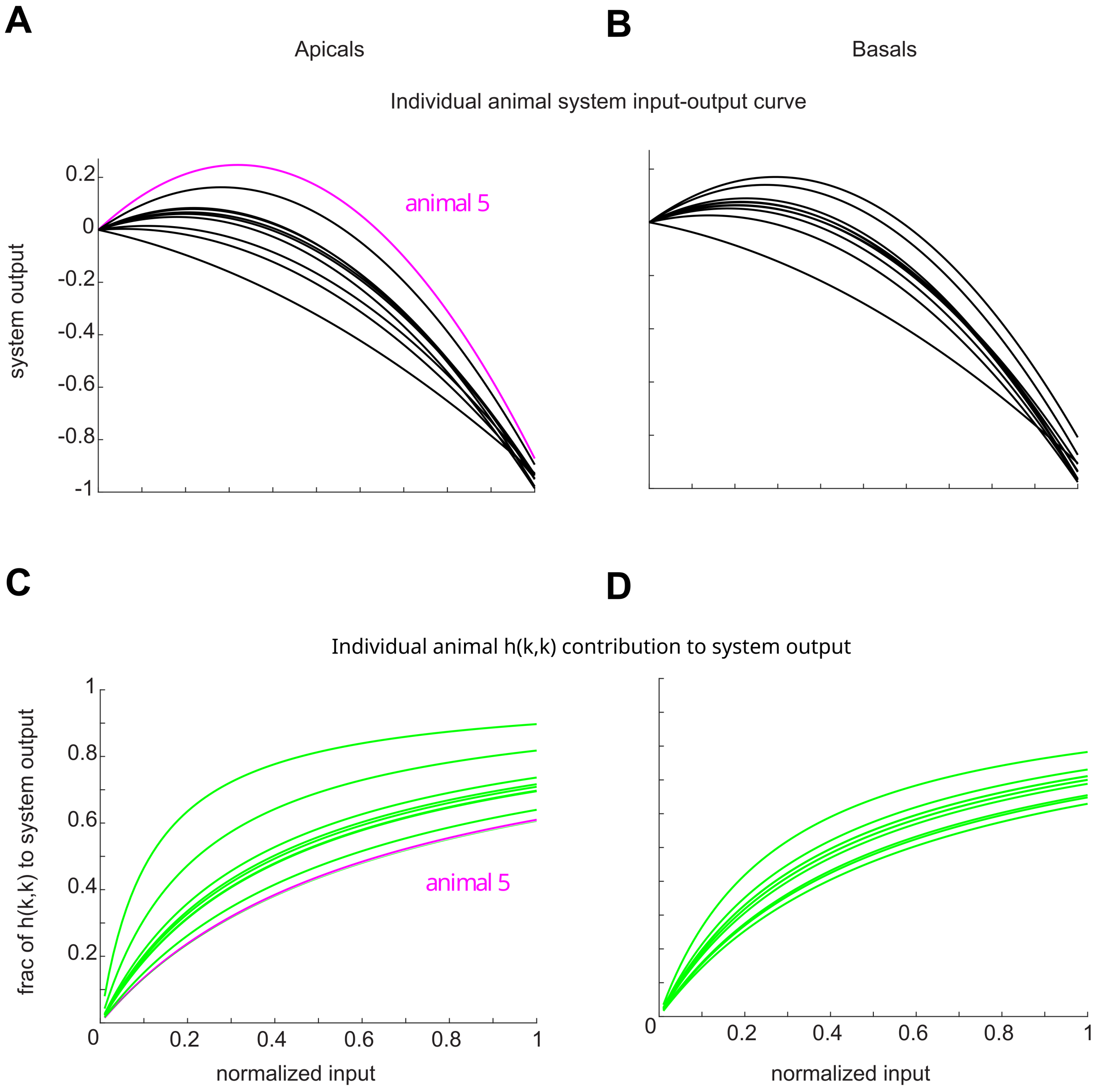

Supplementary Figure 19. Individual animal input-output curves and nonlinear contribution to CA1 output

A-D, Same as in Fig. 4A but plotted separately for (**A-B**) system output and (**C-D**) *h(k,k)* contribution to system output as a function of normalized input. Here, system output is defined as a univariate value (the peak value of the fEPSP) rather than the continuous fEPSP waveform. The timing of the peak (7th msec after stimulation) was identified using the group

level *h(i)* peaks for apicals and basals (Fig. 4A and Fig. 6F, respectively). Left and right panels are for the apical and basal systems, respectively. Note the higher variability across animals for the apicals compared to the basals. Animal 5 is indicated in magenta as an example of different system input-output curves (top left panel) compared to the group. This is a consequence of a highly varied scaling of *h(i)* compared to other animal's estimates (see **Supplementary Figure 13**), and the fact that for this animal, system nonlinearity is only in effect for much larger normalized input values (rightward shift in bottom left panel); this means that such a varied *h(i)* will predominate the output for a larger range of input values, leading to a correspondingly varied input-output curve.

Table 1

Apicals recording table				
Experiment number	Animal number	Slice number	Trial number	Fraction for x_1
1	1	1	1	0.5
2	1	1	2	0.5
3	2	1	1	0.5
4	3	1	2	0.5
5	3	1	1	0.5
6	4	1	1	0.75
7	4	1	2	0.75
8	5	1	1	0.75
9	5	1	2	0.75
10	6	1	1	0.75
11	6	1	2	0.75
12	7	1	1	0.75
13	7	1	2	0.75
14	8	1	1	0.75
15	8	1	2	0.75
16	9	1	1	0.75
17	9	1	2	0.75
18	9	1	3	0.75
19	10	1	1	0.75
20	10	1	2	0.75

Table 1. Apical dendritic recording information regarding each recording session.

Table 2

Basals recording table				
Experiment number	Animal number	Slice number	Trial number	Fraction for x_1
1	1	1	2	0.75
2	1	1	3	0.75
3	2	1	2	0.8
4	2	2	1	0.8
5	2	2	2	0.8
6	2	2	3	0.8
7	3	1	1	0.8
8	3	1	2	0.8
9	3	1	3	0.8
10	4	1	1	0.8
11	4	1	2	0.8
12	4	1	3	0.8
13	5	1	2	0.8
14	5	2	10	0.8
15	6	1	1	0.8
16	6	2	2	0.8
17	6	2	3	0.8
18	6	2	4	0.8
19	6	2	5	0.8
20	7	1	1	0.8
21	7	1	2	0.8
22	7	1	3	0.8
23	7	1	4	0.8
24	8	1	1	0.8
25	8	1	3	0.8
26	9	1	1	0.8
27	9	1	2	0.8
28	9	1	3	0.8
29	9	1	4	0.8
30	9	1	5	0.8
31	9	2	1	0.8
32	9	2	2	0.8
33	9	2	3	0.8
34	9	2	4	0.8
35	10	1	1	0.8
36	10	1	2	0.8
37	10	1	4	0.8

Table 2. Basal dendritic recording information regarding each recording session.
